# Cryo-EM structures of human organic anion transporting polypeptide OATP1B1

**DOI:** 10.1038/s41422-023-00870-8

**Published:** 2023-09-06

**Authors:** Ziyang Shan, Xuemei Yang, Huihui Liu, Yafei Yuan, Yuan Xiao, Jing Nan, Wei Zhang, Wenqi Song, Jufang Wang, Feiwen Wei, Yanqing Zhang

**Affiliations:** 1 Shanghai Fifth People’s Hospital, Fudan University, and Shanghai Key Laboratory of Medical Epigenetics, International Co-laboratory of Medical Epigenetics and Metabolism (Ministry of Science and Technology), Institutes of Biomedical Sciences, Fudan University, Shanghai, China; 2 Warshel Institute for Computational Biology, School of Medicine, The Chinese University of Hong Kong, Shenzhen, Guangdong China; 3 Beijing Frontier Research Center for Biological Structure, Beijing Advanced Innovation Center for Structural Biology, Tsinghua-Peking Joint Center for Life Sciences, School of Life Sciences, Tsinghua University, Beijing, China

**Keywords:** Cryoelectron microscopy, Transport carrier

## Abstract

Members of the solute carrier organic anion transporting polypeptide (OATPs) family function as transporters for a large variety of amphipathic organic anions including endogenous metabolites and clinical drugs, such as bile salts, steroids, thyroid hormones, statins, antibiotics, antivirals, and anticancer drugs. OATP1B1 plays a vital role in transporting such substances into the liver for hepatic clearance. FDA and EMA recommend conducting in vitro testing of drug–drug interactions (DDIs) involving OATP1B1. However, the structure and working mechanism of OATPs still remains elusive. In this study, we determined cryo-EM structures of human OATP1B1 bound with representative endogenous metabolites (bilirubin and estrone-3-sulfate), a clinical drug (simeprevir), and a fluorescent indicator (2′,7′-dichlorofluorescein), in both outward- and inward-open states. These structures reveal major and minor substrate binding pockets and conformational changes during transport. In combination with mutagenesis studies and molecular dynamics simulations, our work comprehensively elucidates the transport mechanism of OATP1B1 and provides the structural basis for DDI predictions involving OATP1B1, which will greatly promote our understanding of OATPs.

## Introduction

**Fig. 1:**
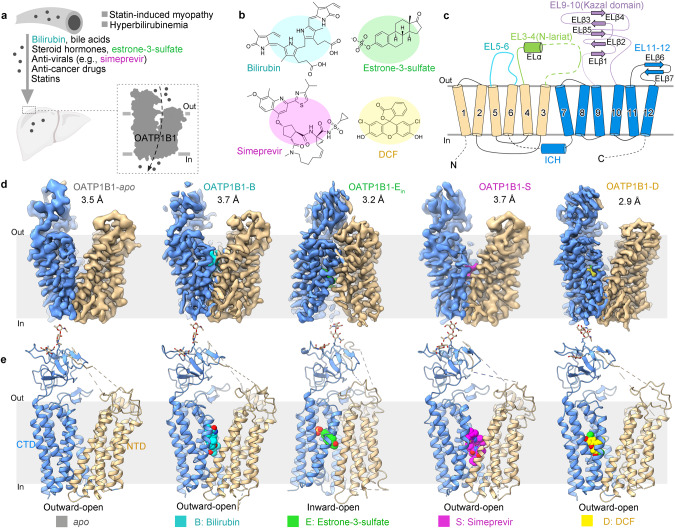
Structure determination and overall structures of human OATP1B1 in the presence of substrates and inhibitor. **a** Schematic representation of the biological functions of OATP1B1. **b** Chemical structures of the four compounds used in this study. **c** The topology of OATP1B1. NTD and CTD are colored in gold and marine, respectively. EL3–4, EL5–6 and EL9–10 are colored in green, cyan and purple, respectively. Dashed lines show the flexible regions in our structures. **d**, **e** Side views of cryo-EM reconstructions (**d**) and cartoon representations (**e**) of human OATP1B1 in apo state and bound with bilirubin, E3S, simeprevir and DCF, colored in cyan, green, magenta, and yellow, respectively. OATP1B1 is shown with NTD and CTD colored in gold and marine, respectively.

Encoded by genes of *SLCO*^1^ solute carrier family (formerly called *SLC21*), the members of the organic anion transporting polypeptides (OATPs) family transport a wide range of amphipathic organic anions across the plasma membrane, including endogenous and exogenous substances, such as bile salts, steroids, thyroid hormones, and clinical drugs.^1,2^ Eleven members of human OATPs have been identified and categorized into 6 subfamilies based on their amino acid composition, namely OATP1–6 (Supplementary information, Fig. S1). Among these OATPs, each member exhibits distinct but partially overlapping substrate specificity, which varies according to their specific tissue distribution.^3^ OATP1B1, encoded by *SLCO1B1* and formerly known as SLC21A6, LST-1, OATP-C, or OATP2, is specifically expressed on the basolateral membrane of hepatocytes.^4^ OATP1B1 plays a vital role in mediating the uptake of endogenous compounds such as bile acids, bilirubin, steroid conjugates, and thyroid hormones, as well as a large variety of clinical drugs including statins, antibiotics, antivirals, and anticancer medications for hepatic clearance (Fig. 1a).^5–7^ Dysfunction of OATP1B1 can lead to statin-induced myopathy^8^ and hyperbilirubinemia,^9^ as exemplified by Rotor syndrome, a type of hereditary hyperbilirubinemia.^10^

Numerous studies have emphasized the central role of OATP1B1 in drug–drug interactions (DDIs).^5,11–13^ Food and Drug Administration and European Medicines Agency recommend conducting in vitro testing to evaluate potential OATP1B1 interactions.^14,15^ Many drugs with significant clinical implications act as substrates and/or inhibitors of OATP1B1.^5^ For instance, in clinical practice, combination therapy involving lipid-lowering statins^16^ (e.g., atorvastatin) and various drugs, such as antihypertensive medications, antibiotics, and antiviral agents, is commonly employed. However, certain medications, such as gemfibrozil, cyclosporine A, rifampicin, and ritonavir, can suppress the function of OATP1B1, leading to excessive systemic exposure of the affected drug, including statins.^11^ The elevation of statin blood levels can potentially lead to fatal myopathy and rhabdomyolysis.^17,18^

The energy coupling transport mechanism of OATPs is still not fully understood. The transport of OATPs is independent of Na^+^, K^+^, Cl^−^ gradients, membrane potential and ATP levels.^19–21^ OATPs are generally believed to act as organic anion exchangers, coupled with the efflux of anions, such as HCO_3_^−^ and glutathione (GSH), but direct evidence was only provided in rat Oatp1a1.^22,23^ However, transport of OATP1B1/OATP1B3 was most probably demonstrated to occur through bidirectional facilitated diffusion, which was unaffected by GSH.^21^ Moreover, evidence has been presented for pH-dependence in transport stimulation for most OATPs with the exception of OATP1C1.^24,25^

To elucidate the transport mechanism of OATP1B1, we determined the structures of OATP1B1 bound with representative endogenous substrates (bilirubin and estrone-3-sulfate (E3S)), a clinical drug (simeprevir), as well as a fluorescent indicator (2′,7′-dichlorofluorescein (DCF)). These structures reveal unexpected major and minor substrate binding pockets and conformational changes between outward-open and inward-open states. Together with mutagenesis functional studies and molecular dynamics (MD) simulations, our work elucidates the transport mechanism of OATP1B1, and provides the structural basis for DDI predictions involving OATP1B1, which will promote the understanding of OATPs.

## Results

### Structure determination

Human OATP1B1 was expressed in Expi293F cells and purified using tandem affinity and size exclusion chromatography. To investigate the transporting mechanism of OATP1B1, we supplemented the protein solution with a saturating concentration of endogenous substrates, bilirubin and E3S, and a clinical drug, simeprevir, during cryo-sample preparation. To better explain the binding mode of the fluorescent probe, DCF, which is employed in the following transport assays, we also prepared the cryo-sample of OATP1B1–DCF (Fig. 1b). The purified OATP1B1 protein alone or supplemented with ligands was then subjected to cryo-electron microscopy (cryo-EM) single particle analysis. For simplicity, OATP1B1 bound with each ligand is hereafter referred to OATP1B1–B (bilirubin), OATP1B1–E (E3S), OATP1B1–S (simeprevir) and OATP1B1–D (DCF).

We determined the structures of OATP1B1-apo/B/S/D in outward-open state at resolutions ranging from 2.9 Å to 3.7 Å (Fig. 1d). The particle dataset of OATP1B1–E contained a mixture of outward-open, inward-open, and occluded states, namely OATP1B1-E_ou_, OATP1B1–E_in_ and OATP1B1–E_oc_, respectively. After extensive trials, we successfully obtained the inward-open structure of OATP1B1–E_in_ at a resolution of 3.2 Å (Fig. 1d; Supplementary information, Fig S5), with OATP1B1–E_ou_ and OATP1B1–E_oc_ resolved at moderate to low resolutions. For details of cryo-EM data acquisition and processing, please refer to Materials and methods (Supplementary information, Figs. S2–S6 and Table S1). The ligand model building was based on the EM densities in a combination of ligand-free docking and MD simulations (Supplementary information, Fig. S7 and Table S2). The relatively small and rigid substances, E3S and DCF, revealed stable binding with OATP1B1, while larger ligands, bilirubin and simeprevir, exhibited more flexibility during MD simulations (Supplementary information, Videos S1–S4).

### Overall architecture

**Fig. 2:**
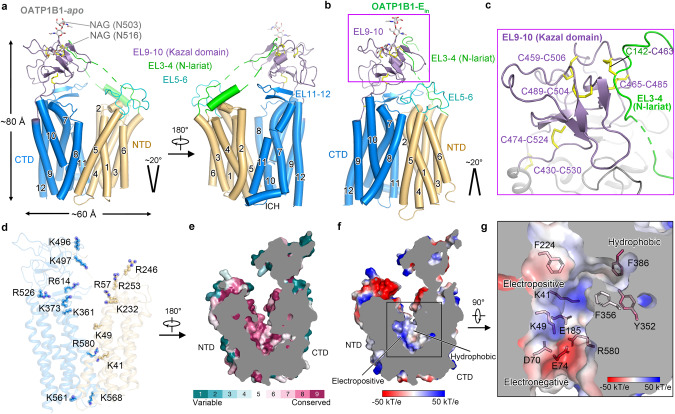
Overall architecture and central cavity environment of OATP1B1. **a** Overall architecture of outward-open OATP1B1-apo. NTD and CTD are colored in gold and marine, respectively. **b** Overall architecture of inward-open OATP1B1–E_in_. **c** Multiple disulfide bonds formed by conserved cysteines within EL9–10 are shown as yellow sticks. A fragment of EL3–4 termed as N-lariat, is attached to EL9–10 via a disulfide bond. **d** Electropositive residues along the translocation path of outward-open OATP1B1-apo. **e** Slab view showing the conservation level of OATP1B1-apo using ConSurf. **f** Sliced view of the surface electrostatic potential of OATP1B1-apo. **g** Top view of key residues in the central cavity of outward-open OATP1B1-apo, forming electropositive, electronegative, and hydrophobic parts. Residues are shown as stick and colored based on ConSurf analysis.

The overall architecture of OATP1B1 is composed of the transmembrane domain (TMD) and extracellular loops (ELs) with both N- and C-terminal ends situated in the cytoplasm. The TMD follows the canonical MFS fold^26^ and consists of 12 transmembrane helices (TMs) organized into an N-terminal domain (NTD) and a C-terminal domain (CTD), exhibiting a 2-fold pseudo symmetry around an axis perpendicular to the membrane plane (Fig. 1c). The NTD and CTD are connected by an intracellular helix (ICH) (Fig. 2a, b).

The largest extracellular loop, EL9–10, features several β sheets and pairs of disulfide bonds, known as the Kazal domain^27^ (Fig. 2c). The Kazal domain is an evolutionarily conserved protein domain, usually indicative of serine protease inhibitors,^28^ but its function in OATPs still remains elusive. Within EL9–10, two glycosylation sites (N503 and N516) were observed to be covalently linked to N-acetylglucosamine sugar moieties (Fig. 2a, b). In both outward- and inward-open states of OATP1B1, EL3–4 associates with the Kazal domain through a conserved disulfide bond C142–C463, designated as N-lariat (Fig. 2c). MD simulations of the outward-open states with EL3–4 disconnected resulted in abnormally wider opening (data not shown), indicating that the N-lariat potentially regulates the maximum opening extent and acts as an extracellular halter connecting the extracellular halves.

### Substrate entrance and central cavity environment

The translocation path is lined with multiple basic residues (Fig. 2d). An extracellular electropositive opening, enclosed by EL5–6, the Kazal domain and N-terminus of TM8, potentially functions as an entrance for organic anion substrates to access the central cavity (Supplementary information, Fig. S8a, b). Most of the positively-charged residues are distributed at this indicated entrance rather than in the cavity itself, implying that selectivity for organic anions might be achieved by partially filtering out cations at the cavity entrance. Besides, in the outward-open OATP1B1-apo/B/S/D, lipid-like EM densities were trapped in the V-shaped lateral opening formed by the two halves between TM2 and TM11 (Supplementary information, Fig. S8c), which might serve as another potential access for hydrophobic substrates, as described in OCT3.^29^

**Fig. 3:**
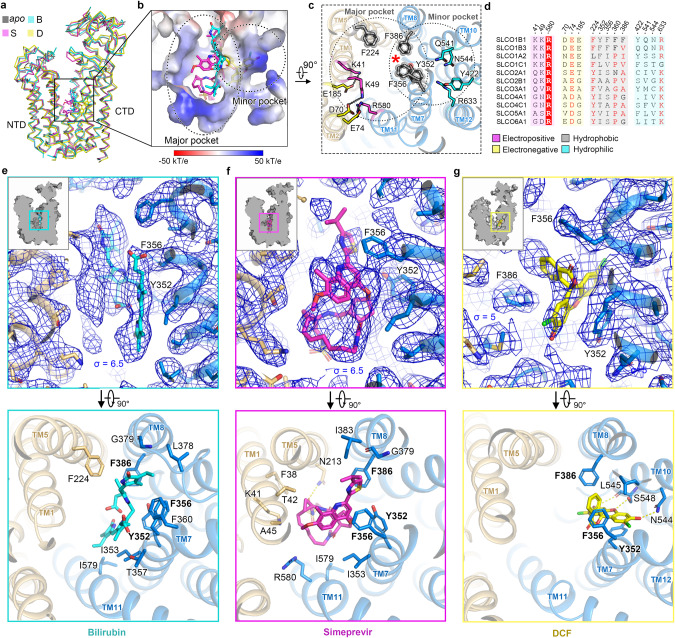
Substrate binding sites in the outward-open state. **a** Superimposition of outward-open structures of OATP1B1-apo/B/S/D. **b** Bilirubin and simeprevir occupy the major pocket and DCF occupies the minor pocket, which is superimposed and presented in the central cavity of OATP1B1–D. **c** Top view of outward-open OATP1B1-apo showing key residues in the major pocket and minor pocket. Electropositive residues, electronegative residues, hydrophobic residues, and hydrophilic residues are colored in magenta, yellow, gray and cyan, respectively. **d** Sequence alignment of key residues in the central cavity of OATP1B1 among SLCO family transporters. **e**–**g** Local ligand density representations and extracellular views for substrate binding sites of OATP1B1–B (**e**), OATP1B1–S (**f**) and OATP1B1–D (**g**) in outward-open state.

The interface between NTD and CTD of OATP1B1 forms a conserved cavity in which substrates bind (Fig. 2e). The central cavity exhibits evident features of both hydrophobicity and electro-positivity, wherein TMs 5, 7, 8, and 10 contribute to the hydrophobic side while TMs 1, 2, 4, and 11 form the charged side partly localized oppositely to the hydrophobic side (Figs. 2f, g, and 3c). Positively-charged residues, K41/K49/R580, along with modestly conserved aromatic residues, i.e., Y352, F356, and F386, create an ideal environment for selecting and translocating amphipathic organic anion substrates within the central cavity. Of note, all three basic residues participate in generating charge pairs, K41–E185, K49–D70 and highly conserved R580–E74 (Figs. 2e, g and 3c, d).

### Substrate binding sites in outward-open state

Bilirubin is a representative endogenous substrate of OATP1B1, which is a kind of biologically toxic metabolite produced as a byproduct of heme degradation in red blood cells. Impaired elimination of bilirubin can lead to hyperbilirubinemia, which is closely associated with multiple disorders and reflects abnormal liver function.^30^ As for simeprevir, a direct-acting antiviral drug for the treatment of hepatitis C virus infection, it not only acts as a substrate for OATP1B1 but also functions as an inhibitor.^31,32^ Simeprevir can lead to clinical DDIs, as exemplified by an approximately 2-fold increase in plasma when administered concomitantly with atorvastatin or rosuvastatin.^32^ DCF is a fluorescein derivative and has been previously identified to be a good substrate and probe to assess the transport activity of OATP1B1.^33–37^

Structures of OATP1B1 bound with bilirubin, simeprevir and DCF, adopt similar outward-open conformations as that of OATP1B1-apo (Fig. 3a). The root mean square deviation between OATP1B1-apo and each of the ligand-bound states is 0.51 Å (apo vs bilirubin), 0.46 Å (apo vs simeprevir), and 0.89 Å (apo vs DCF), respectively. Typically, MFS transporters engage TMs 1, 4, 7, 10 (known as A helices) to bind ligands.^26,38^ However, OATP1B1 also employs B helices to bind substances, i.e., with TMs 5, 7, 8, and 11 to bind with bilirubin, TMs 1, 5, 7, 8, and 11 to bind with simeprevir, and TMs 7, 8, and 10 to bind with DCF.

Based on the ligand binding sites, two distinct pockets were observed within the translocation pathway. Bilirubin and simeprevir occupy the major pocket located in the central cavity, while DCF binds to the minor pocket extending into the NTD (Fig. 3b). The binding of these ligands primarily relies on interactions with hydrophobic residues opposite to the charged side in the central cavity (Fig. 3c, e–g; Supplementary information, Fig. S7). B/S/D are all closely attached to F356 and Y352 around the middle of TM7, further clamped by F386 from TM8, acting as a core binding hub. The EM densities of ligands of B/S/D were observed to clearly adhere to F356 (Fig. 3e–g). However, no obviously direct associations were observed between ligands and the positively-charged residues in the central cavity.

The multiple residues interacting with bilirubin are mostly derived from the CTD, enabling bilirubin to closely associate with the hydrophobic side in the central cavity. Comparably, simeprevir engages in more interactions with residues from both the NTD and CTD, wherein F356 forms hydrophobic packing with the quinoline group of simeprevir. In vitro studies have shown that simeprevir can inhibit the transport of multiple substrates of OATP1B1.^31,39^ The characteristic of larger spatial occupation and more complex interactions that simeprevir presented, is in accord with those of previously characterized OATP1B1 inhibitors manifesting significantly large molecular weight and high lipophilicity.^40,41^

Different from the binding mode of bilirubin and simeprevir, the main body of DCF extends towards a minor hydrophilic pocket within the interior of the CTD by forming hydrogen-bond interactions with N544 and S548. Meanwhile, residues from the core binding hub, Y352, and F356 form hydrophobic packing with the xanthen and benzofuran groups of DCF, respectively (Fig. 3g; Supplementary information, Fig. S7d). The co-existence of major and minor pockets is consistent with the assumption that multiple substrate binding sites are in OATP1B1.^13,42^

We further performed DCF uptake inhibition assay with mutagenesis to validate the ligand poses built in the structures of OATP1B1–B/S. Consistent with the poses that we built, K49A, F360A, and I383A exhibited substrate-dependent inhibition despite slight variations in their IC_50_ values for bilirubin and simeprevir, respectively (Supplementary information, Fig. S9).

### E3S-bound OATP1B1 adopts an inward-facing conformation

**Fig. 4:**
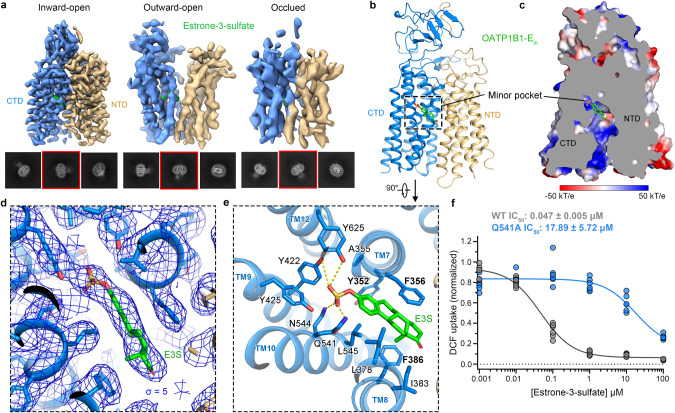
Inward-facing conformation of OATP1B1 in complex with E3S. **a** Three different conformations were captured when OATP1B1 is supplemented with E3S. Shown here are cryo-EM reconstructions (upper) and representative 2D class averages (lower) of OATP1B1 bound to E3S that correspond to inward-open, outward-open, and occluded conformations, respectively. 2D class average boxed in red shows the representative view in each conformation. Owing to the moderate to low resolutions of the outward-open and occluded conformations, no structural model was assigned. **b** Cartoon representation of OATP1B1–E_in_ showing the binding site of E3S in the overall structure. **c** Slab view of the electrostatic surface of E3S binding site, wherein E3S binds in the minor pocket. **d** Local EM density map of E3S in OATP1B1–E_in_ contoured at 5 σ. **e** Expanded view of E3S binding site in OATP1B1–E_in_. **f** Inhibition of OATP1B1 transport by E3S (mean ± SEM, *n* = 6). *n* = 6 represents six biologically independent experiments for each cell line.

Compared to lipophilic estrone and estradiol, the estrogen ester and conjugate, E3S, is unable to permeate through cell membranes and is transported mainly via OATPs, which has been widely used as a prototypical model substrate of OATP1B1.^43–45^ Inhibitors of OATPs are of interest in treating estrogen-associated cancers.^46^ In our study, the cryo-EM structures of OATP1B1–E unexpectedly revealed three different conformations: inward-open state with high resolution, outward-open and occluded states with moderate to low resolutions (Fig. 4a). Densities of E3S were found in all three conformations at similar sites (Supplementary information, Fig. S10). The EM density of E3S in OATP1B1–E_in_ is unambiguously resolved, allowing us to discern the pose of the E3S with high confidence (Fig. 4d; Supplementary information, Fig. S7b).

**Fig. 5:**
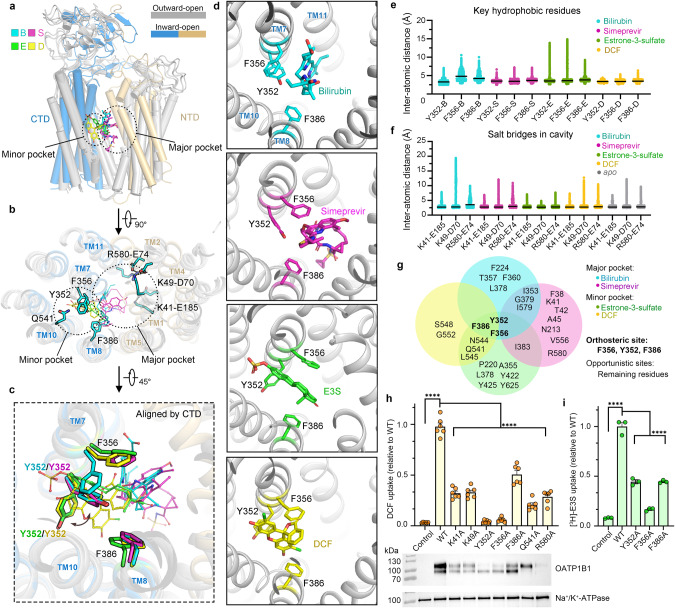
Structural determinants for broad substrate specificity. **a** Side view of the structural alignment of the outward-open and inward-open structures of OATP1B1 relative to the membrane norm. **b** Extracellular view showing key residues and three pairs of salt bridges in the central cavity. Four compounds in this study together occupy both the major pocket and minor pocket. Overall structures are aligned relative to membrane norm. **c**, **d** Extracellular views of four compounds with overall structures aligned by CTD (**c**) and four individual compounds (**d**), showing three key residues in substrate binding sites, Y352, F356 and F386. **e** The distances between center of mass of ligands and sidechains of key residues forming the central binding hub, Y352, F356 and F386 in 500-ns MD simulations, showing the stable interactions between the three residues and ligands. **f** The distances between sidechain of residues in each pair of salt bridge in 500-ns MD simulations of all the structures in our study, showing the stable interactions within salt bridges. **g** Schematic summary of key residues involved in the binding of four compounds in our study, showing orthosteric and opportunistic sites. **h** DCF uptake activity assay showing reduced activity of OATP1B1 mutants regarding residues within major and minor pockets. The activity was normalized to the wild-type (WT) transporter (mean ± SEM, *n* = 6). Control indicates empty cell control. Surface expression levels are shown in western blotting for control, WT and mutants. **i** Liposome assay of [^3^H]-E3S uptake showing reduced activity of OATP1B1 mutants regarding three key residues forming the central binding hub. The activity was normalized to the WT transporter (mean ± SEM, *n* = 3). Control indicates empty liposome control.

The inward-open structure of OATP1B1–E_in_ opens its central substrate cavity towards the cytoplasm (Fig. 4b, c). Like DCF, E3S also binds at the minor pocket, with its sulfate group extending into the interior of the CTD (Fig. 4b, c). F356 forms hydrophobic packing with the estrone group of E3S, together with Y352 and F386, enclosing the hydrophobic body of E3S (Fig. 4e; Supplementary information, Fig. S7b). Additionally, residues from the minor pocket, Y625, Y422, Q541 and N544 form hydrogen-bond interactions with the sulfate group of E3S. Mutagenesis of Q541A exhibited a significantly varied IC_50_ value for E3S, supporting the essential role of Q541 for the binding of E3S (Fig. 4f). Accordingly, Q541A led to an evident decrease of DCF uptake activity (Fig. 5h; Supplementary information, Fig. S12). Of particular note, like DCF, the residues involved in the binding of E3S are basically derived from the CTD.

### Structural determinants for broad substrate specificity

The ligands, bilirubin and simeprevir, occupy the central cavity in the major pocket in the outward-open state, while DCF and E3S are located at the minor pocket in the outward- and inward-open conformations, respectively (Fig. 5a, b). Superposition of OATP1B1-apo/B/S/D/E structures reveals rearrangements of Y352 and F356 from TM7 upon binding of different ligands. By undergoing a vertical rotation of the side chain of Y352, smaller substrates, i.e., DCF/E3S, can be accommodated in the minor pocket towards the interior of the CTD (Fig. 5c, d).

Each of the ligands (B/S/E/D) exhibits distinct but partially overlapping binding sites with OATP1B1. The hydrophobic mass centers of these substrates are all associated at the core binding sites, composed of three aromatic residues, Y352, F356 and F386 (Fig. 5b–d). [^3^H]-E3S liposome assay and cell-based DCF uptake assay confirmed the essential roles of these binding hub residues in transport, especially Y352 and F356 (Fig. 5h, i). The stable binding of ligands to Y352, F356 and F386 during 500-ns MD simulations in three replicates further indicates the critical role of these residues in substrate binding (Fig. 5e; Supplementary information, Fig. S11a–d and Videos S1–S4). We term the binding hub formed by the three aromatic residues as the orthosteric site. Additionally, the central cavity possesses a significant spatial volume, allowing large compounds to bind effectively within the primary pocket. Each of the ligands also possesses their own special binding sites in the major or minor pocket, which we term as opportunistic sites, as recently described in OCT1^47^ (Fig. 5g).

OATP1B1-interacting substrates and drugs are structurally diverse with only nebulous similarities, i.e., greater hydrophobicity, higher complexity, and more ringed structures.^48^ The orthosteric site of OATP1B1 might play a pivotal role in hydrophobic packing with the fairly hydrophobic compounds. The combination of the orthosteric site and the opportunistic site in both major and minor pockets, might collectively contribute to the substrate poly-specificity of OATP1B1. Similarly, the corresponding residues in the orthosteric site of OATP1B3 are F352 and F356, potentially leading to the similar function and substrate specificity between OATP1B1 and OATP1B3.^1^ On the contrary, in other OATPs with relatively distinctive substrate range and tissue distribution,^3^ the three aromatic residues are less conserved and mainly substituted by smaller hydrophobic residues (Fig. 3d; Supplementary information, Fig. S1), indicating that the residues in the orthosteric site may determine the substrate specificity of different OATPs.

Of note, despite OATP1B1 substrates being primarily organic anions, no apparent interactions were observed between ligands and the basic residues, K41/K49/K580, within the cavity of OATP1B1–B/S/E/D. As observed in our structures, MD simulations confirmed the presence of three charge pairs: K41–E185, K49–D70 and K580–E74 in the cavity (Fig. 5f), suggesting that the positively-charged residues might contribute to modulating the electrostatics of the cavity rather than direct interacting with anion substrates. The transport activities of K41A, K49A, and R580A exhibit evident reduction despite decreased surface expression, especially R580, suggesting their vital roles (Fig. 5h). Consistently, previous studies have shown that E74A/Q and R580K led to decreased transport activity of OATP1B1.^49,50^

### Rocker-switch alternating access of transport

**Fig. 6:**
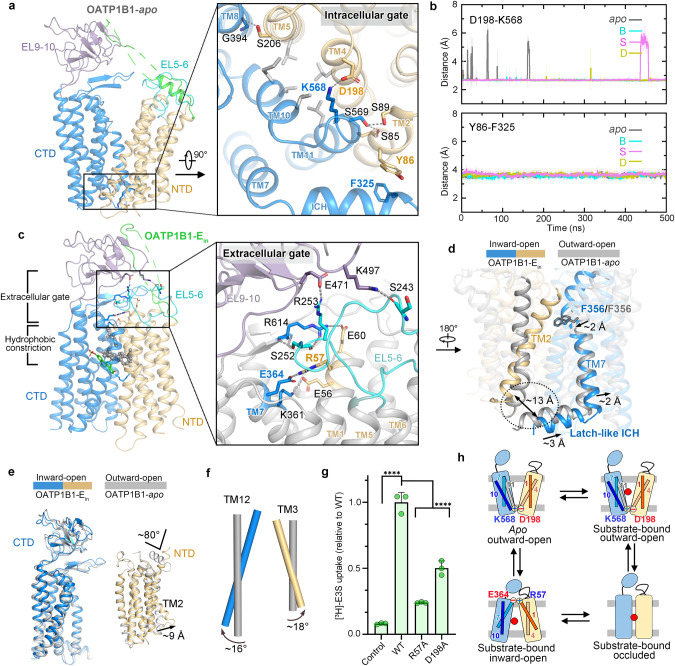
Rocker-switch alternating access of transport. **a** The intracellular gate in the outward-open state. **b** Distances of residues involved in intracellular gate in outward-open OATP1B1-apo/B/S/D in MD simulations. Upper panel: the distances between center of mass of D198 sidechain carboxyl group and K568 sidechain N atom. Lower panel: the distances between center of mass of Y86 and F325 sidechain. **c** An extracellular gate and hydrophobic constriction in inward-open OATP1B1–E_in_. Residues forming the hydrophobic constriction are shown in gray sphere. The extracellular gate is magnified in the right panel. **d** Dissociation of latch-like ICH and TM2 during transition from outward-open to inward-open state relative to membrane norm. **e** Structural comparison between inward-open and outward-open structures relative to CTD and NTD separately. **f** Rotation of representative TM segments between inward-open and outward-open structures relative to the membrane norm. **g** Liposome assay of [^3^H]-E3S uptake showing reduced activity of OATP1B1 mutants regarding key residues in intra- and extracellular gates. The activity was normalized to the WT transporter (mean ± SEM, *n* = 3). Control indicates empty liposome control. **h** Rocker-switch alternating access model of OATP1B1 during transport. Conformations of OATP1B1 that are achieved in this study are shown. The red circle represents substrate.

The intra- and extracellular gates are formed in the outward- and inward-open state, respectively. In outward-open OATP1B1-apo/B/S/D, the intracellular gate is closed mainly by TM4 and TM10. A conserved salt bridge is formed by the interaction between D198 and K568 (Fig. 6a). Additionally, multiple hydrophobic residues act as barriers, blocking the intracellular central pathway. Particularly, the ICH is attached to the C-terminus of TM2 through hydrophobic interactions between F325 and aromatic residues, F83, Y86 and F87. This attachment potentially contributes to the sealing and maintenance of the intracellular gate in the outward state (Fig. 6a). MD simulations support the stable existence of both D198–K568 salt bridge and Y86–F325 hydrophobic interaction in outward-open OATP1B1-apo/B/S/D (Fig. 6b; Supplementary information, Fig. S11e, f). Mutagenesis of D198A significantly decreased the [^3^H]-E3S uptake of OATP1B1, indicating its vital role during transport (Fig. 6g). Similarly, mutations of Y86A, D198A and K568A almost completely abolished the transport activity of DCF, as determined by confocal microscopy (Supplementary information, Fig. S12).

In the inward-open OATP1B1–E_in_, accompanying the disappearance of the intracellular gate, an extracellular gate is generated by electrostatic interactions between NTD and CTD (Fig. 6c). Two charge pairs, R57–E364 and E56–K361, originating from TM1 and TM7, respectively, are highly conserved. In addition, electrostatic interactions of R614–E60, E471–R253 and K497–S243 further contribute to the closure of extracellular gate. Mutagenesis of one highly conserved salt bridge, R57A/E364A, resulted in significant decrease in the uptake of [^3^H]-E3S or DCF, suggesting their essential roles during transport (Fig. 6g; Supplementary information, Fig. S12).

Interestingly, DCF and E3S both bind at the minor pocket, while they arrest OATP1B1 in outward-open and inward-open states, respectively, indicating a mechanism of substrate-induced conformational changes. Connected with TM7 like an elbow, ICH closely associates with TM2 like a latch in the outward-open state (Fig. 6a, d). Upon binding of E3S, the fine-tuning of F356 in the orthosteric site potentially triggers the synergic movement of TM7 and the latch-like ICH, followed by the dissociations of TM2b and ICH and conformational alteration of TM2b from a bent to a straight form, ultimately leading to the opening of intracellular gate and sequentially global conformational changes (Fig. 6d; Supplementary information, Fig. S13). Additionally, the local adjustment of F386 and L378 upon binding of E3S causes a slightly dilated movement of TM8, probably disrupting the hydrophobic interactions of F391–I211 and Y395–L207 (Supplementary information, Fig. S13d). A hydrophobic constriction network was identified above E3S, wherein the estrone group of E3S is engaged in hydrophobic interactions with F356, I378, F224, and I46, potentially facilitating the conformational transition from the outward-open to the inward-open state while DCF induces no such interactions (Fig. 6c; Supplementary information, Fig. S13c).

When we compare the inward- and outward-open states with respect to NTD or CTD separately, the overall structure of each domain remains nearly unchanged (Fig. 6e). In general, substrate translocation by OATP1B1 is achieved through rigid-body rotations of the NTD and CTD that expose the central substrate cavity alternatively to extracellular or cytosolic side of the membrane, conforming to the rocker-switch model of MFS transporters (Fig. 6e, f, h; Supplementary information, Video S5).^26,51^ It is worth noting that the highly conserved charge pair R580–E74, located around the middle of TMD and originating from two halves, might play a role of acting as a central pivot during conformational changes (Fig. 5f; Supplementary information, Fig. S11g).

## Discussion

In this study, we successfully resolved the structures of OATP1B1 in different states, including substrate-free outward-open state, substrate-bound outward-open state, substrate-bound inward-open state, and substrate-bound occluded state at low resolution, adopting rocker-switch model during transport (Fig. 6h). Nevertheless, despite the states we captured, further intermediate states, i.e., outward-occluded or inward-occluded, might exist,^51^ which needs further investigation.

The energy coupling transport mechanism of OATPs is not fully understood yet. Evidence for pH-dependence in transport stimulation has been shown in most OATPs except for OATP1C1.^24,25^ The pH-dependence of OATP1B1 seems substrate-dependent, given that the transport of E3S was not affected by pH.^21,24^ A conserved histidine among OATPs, located at the EL5–6, is replaced by a glutamine in OATP1C1, which was found to diminish its pH-sensitivity of transport.^24^ The structure of OATP1B1–B/S/E/D in our study revealed that this conserved H115 directly interacts with D236, which might enhance the rigid-body movement of EL5–6 with the NTD transmembrane bundle, partially explaining the potential facilitative function of H115 for the pH-sensitive transport (Supplementary information, Fig. S14). Moreover, considering a series of extracellular charge pairs in the inward-open state of OATP1B1, the pH-dependence might facilitate the opening of the extracellular gate during transport. However, previous transport assays investigating the energetics of OATPs have predominantly employed *Xenopus laevis* oocytes or human cell lines. A significant issue arises from the lack of in vitro liposome assays to validate the energetics. The in vitro [^3^H]-E3S liposome assay that we performed indicated that the chemical concentration of substrate can probably act as driving force for the transport of OATP1B1 (Figs. 5i, 6g), in accord with the previously proposed bidirectional facilitated diffusion of OATP1B1/OATP1B3.^21^ The coupling energy mechanism might not be identical in OATPs and further research is needed to fully understand the driving forces and regulatory factors involved.

The cellular uptake of a broad range of structurally diverse organic anions and cations mainly depends on two SLC transporter families, OATPs and SLC22A superfamilies which contain the organic cation transporters (OCTs) and the organic anion transporters (OATs).^52^ In general, OCTs transport cations, OATPs transport large and fairly hydrophobic organic anions, and OATs transport the smaller and more hydrophilic organic anions. According to the recently published structures of OCT1–3^29,47^ and rat Oat1,^53^ the substrate binding pocket is highly electronegative or electropositive, creating a selective pathway for the translocation of organic cations or organic anions, respectively.^29,47^ Overlapping and distinct binding site, or termed as the orthosteric site and the opportunistic sites, were also observed in OCTs and rat Oat1 when bound to different ligands, consistent with their poly-specific nature of substrate recognition similar to OATP1B1. The central pockets of OATs and OCTs are relatively smaller compared to OATP1B1, explaining why OATPs tend to transport larger substrates while OCTs and OATs mostly transport smaller compounds (Supplementary information, Fig. S15).

In summary, our work presents the major and minor substrate binding sites of OATP1B1 and conformational changes during transport, elucidates the broad substrate specificity and transport mechanism of OATPs and provides crucial structural reference for guiding combination therapy strategies and future drug design to prevent DDIs involving OATPs.

## Materials and methods

### Protein expression and purification

cDNA of human *OATP1B1* (UniProt ID: Q9Y6L6) gene was synthesized and cloned into a pCAG vector containing a C-terminal tandem His and Flag affinity tags. For the expression of OATP1B1 protein, Expi293F cells were used and cultured in SMM 293T-II medium at 37 °C under 5% CO_2_ at 130 rpm in a Multitron-Pro shaker. When the cell density reached approximately 2.0 × 10^6^ cells/mL, Expi293F cells were transiently transfected with the plasmid and polyethylenimines (PEIs) (Polysciences). The total amount of plasmids for transfection of one liter of cell culture is 2 mg and plasmids were pre-mixed with PEIs in 40 mL fresh medium in a 1:2 (w/w) ratio for 15 min before adding the mixture into cell culture.

48 h after transfection, cells were harvested by centrifugation at 2000× *g* and resuspended in the buffer containing 25 mM HEPES pH 7.4, 150 mM NaCl, 1.3 μg/mL aprotinin (Amresco), 0.7 μg/mL pepstatin (Amresco), and 5 μg/mL leupeptin (Amresco). The membrane fraction was extracted with 2% (w/v) n-Dodecyl-β-D-Maltopyranoside (DDM, Anatrace) at 4 °C for 2 h. After extraction, the cell lysate underwent centrifugation at 12,000× *g* for 1 h (JA 25.50, Beckman Coulter) and supernatant was loaded to anti-Flag M2 affinity resin (GenScript), washed in buffer containing 25 mM HEPES pH 7.4, 150 mM NaCl, 0.01% LMNG (w/v), and eluted with buffer containing 25 mM HEPES pH 7.4, 150 mM NaCl, 0.01% LMNG (w/v) and 0.4 mg/mL Flag peptide. The protein sample after elution was concentrated and further purified by size-exclusion chromatography on a Superose 6 10/300 GL column (GE Healthcare) in the above wash buffer. The protein peak fraction was determined by SDS-PAGE and collected for cryo-EM analysis.

### Cryo-EM sample preparation and data acquisition

The final concentration of protein samples for cryo-EM sample preparation is ∼5–10 mg/mL. For OATP1B1–B/E/S/D, compounds of bilirubin (TargetMol), estrone sulfate sodium (MedChemExpress), simeprevir (MedChemExpress), and 2’,7’-dichlorofluorescein (Sigma-Aldrich) were added to the protein solution at a saturating concentration of 190 μM, 2.6 mM, 440 μM and 1.2 mM, individually. 4 μL of protein sample with compounds was applied to a glow-discharged Quantifoil R1.2/1.3 300-mesh gold holey carbon grid, blotted for 4–5 s and flash-frozen in liquid ethane cooled by liquid nitrogen with Vitrobot (Mark IV, Thermo Fisher Scientific).

For data acquisition of OATP1B1-apo/B/S, micrographs were acquired on a Titan Krios microscope (Thermo Fisher Scientific) operated at 300 kV with a K3 Summit direct electron detector (Gatan) and a GIF Quantum energy filter. SerialEM^54^ software was used for automated data collection following standard procedures. A nominal magnification of 105,000× was used for imaging, yielding a pixel size of 0.832 Å on images. The defocus range was set from −1.0 μm to −1.8 μm. Each micrograph was dose-fractionated to 40 frames in a total dose of ∼50 e^–^/Å^2^, with total collection of 12,329 and 14,613 and 8266 movie stacks, respectively, for OATP1B1-apo/B/S.

For data acquisition of OATP1B1–E/D, micrographs were acquired on a Titan Krios microscope (Thermo Fisher Scientific) operated at 300 kV with a Falcon 4i Direct Electron Detector (Thermo Fisher Scientific) and a Selectris X energy filter. EPU was used for automated data collection following standard procedures. A nominal magnification of 130,000× was used for imaging, yielding a pixel size of 0.932 Å on images. The defocus range was set from –0.9 μm to –1.5 μm. Each micrograph was recorded in a total dose of ∼50 e^–^/Å^2^, with total collection of 13,425 and 9687 movie stacks, respectively for OATP1B1–E/D.

### Cryo-EM data processing

The data processing pipelines are shown in Supplementary information, Figs. S2–S6. Motion correction and CTF estimations were performed using cryoSPARC v3.3.2.^55^

For the data processing of OATP1B1-apo, after several rounds of 2D classification and Ab-initio reconstruction using cryoSPARC, an initial model with clear TMs was obtained. Heterogeneous Refinement, Local refinement and Local CTF refinement yielded an outward-open OATP1B1-apo at the resolution of 3.53 Å. During the data processing, “seed”-facilitated 3D classifications using cryoSPARC and skip-align 3D classification via RELION v3.1^56^ contributed to resolution improvement (Supplementary information, Fig. S2 and Table S1).

The initial models generated from good 2D classes in datasets of OATP1B1–B/S/D were very similar to that of OATP1B1-apo, all in outward-open conformations. Therefore, the cryo-EM structure of OATP1B1-apo was used as initial reference. To eliminate reference bias, initial models were re-generated from good particles using Ab-initio in cryoSPARC in the following data processing. Heterogeneous Refinement, Local refinement and Local CTF refinement were applied. 3D classification improved the density for ligands. Outward-open structures of OATP1B1–B/S/D were achieved at the resolution of 3.73 Å, 3.73 Å, and 2.92 Å, respectively (Supplementary information, Figs. S3, S4, S6 and Table S1).

During the data processing for OATP1B1-E, distinct conformations were observed in the generated initial models from good 2D classes. Data processing using the OATP1B1-apo as the initial reference only resulted in an outward-open OATP1B1–E at the resolution of 4.4 Å. Except for outward-open conformation, the occluded and inward-open conformations of OATP1B1–E were also captured at low resolution. Alternant initial model generation and “seed”-facilitated 3D classifications contributed to reconstructing the inward-open OATP1B1–E_in_ at the resolution of 3.19 Å (Supplementary information, Fig. S5 and Table S1).

### Model building

We first built the atomic model of OATP1B1-apo based on the predicted model generated by SWISS-MODEL^57^ using Protein Data Bank ID: 7EEB as a starting template. Atomic model building based on the density map of OATP1B1-apo was performed in Coot,^58^ refined in real space using Phenix.real_space_refine.^59^ Validation tools in Phenix^59^ and Molprobity^60^ were used to guide iterative rounds of model adjustment in Coot and refinement in Phenix. Since the conformations of OATP1B1–B/S/D were very similar to that of OATP1B1-apo, atomic model of OATP1B1-apo was used as initial model for model building of OATP1B1–B/S/D, followed by manual adjustment to fit into density maps. For the model building of OATP1B1–E_in_, AlphaFold^61,62^ predicted model with an inward-open conformation conforms to the density map well, with subsequent modifications in accord with the density map.

We performed molecular free docking and estimated the binding free energies using the molecular mechanics-generalized Born surface area (MM/GBSA) with the Schrödinger Maestro program to determine the ligand binding pose. Then we built the proposed model of the ligands based on the EM density map and surrounding environment using Coot, followed by adjustment with the assistance of Phenix.real_space_refine and MD simulations. All figures were prepared in PyMoL (www.pymol.org) or UCSF ChimeraX.^63^

### MD simulations

MD simulations were conducted for both OATP1B1-apo protein and four protein–ligand complexes, OATP1B1–B/S/E/D. The initial proteins include residues 25–651 for outward-open conformation and residues 26–652 for inward-open conformation. Charged residues were protonated assuming pH 7.4, and the N- and C-termini of proteins were capped with acetyl and N-methyl groups, respectively. To build the system, proteins or protein–ligand complex was firstly embedded in a POPC bilayer (~350 molecules) and then solvated in a water box (128 × 128 × 125 Å^3^). Finally, 150 mM NaCl was added to neutralize the system. Details of system composition are listed in Supplementary information, Table S2.

MD simulations were performed with GROMACS 2021.4.^64^ CHARMM36 force field was used for protein, POPC lipid and ions,^65,66^ and TIP3P model^67^ was used for water. The force fields of four ligands (bilirubin, simeprevir, E3S, and DCF) were generated by CgenFF program.^68,69^ After energy minimization by steepest descent algorithm, the systems were simulated in NVT ensemble to relax lipid tail, lipid head and solvent (water and ions) in steps for 1.5 ns. Then, the systems were further relaxed in NPT ensemble. Constraints on (1) lipids and solvent, (2) ligand (if any), (3) protein sidechain and (4) protein Cα atoms were gradually released each for 2 ns. After pre-equilibrations, 500 ns production simulations were run without any constraint. Periodic boundary condition was applied, and the temperature and pressure were kept at 310 K and 1 atm by Nose-Hoover thermostat^70,71^ and Parrinello-Rahman barostat,^72^ respectively. Long-range electrostatic interactions were described using the particle mesh Ewald (PME) method^73^ with a cutoff of 12 Å. Van der Waals interactions were smoothly switched to zero from 10 Å to 12 Å. All bonds involving hydrogen atoms were constrained by the SHAKE algorithm^74^ to allow a time step of 2 fs. Each system was run for three replicates with different initial velocities and the total simulation time was 7.5 µs.

### OATP1B1 transport activity assay using DCF

The uptake activity of OATP1B1 WT and mutants was measured by DCF influx using HEK293T cells. The transport activity of OATP1B1 was determined by DCF uptake of cells overexpressing OATP1B1 proteins. Cells cultured in a 6-well plate were used for transport measurement. To each well, HEK293T cells were transfected with pCAG-OATP1B1-His-Flag constructs, using Lipofectamine 2000 reagent (Invitrogen) following the manufacturer’s instructions and the total amount of DNA per transfection was 2.5 μg. Cells transfected with PBS instead of plasmids were used as blank control. After 48 h, DMEM medium in the dish was aspirated out and cells were washed with pre-warmed Phosphate-Buffered Saline (PBS) for three times. Then, 1 mL pre-warmed uptake buffer (142 mM NaCl, 5 mM KCl, 1 mM KH_2_PO_4_, 1.2 mM MgSO_4_, 1.5 mM CaCl_2_, 5 mM D-glucose, 12.5 mM HEPES pH 7.4) with 5 μM DCF dissolved in was added to each well. After incubation for 5 min at 37 °C, uptake buffer with DCF was aspirated out and cells were washed with pre-cooling PBS for three times. Then, 500 μL uptake buffer with 2% Triton X-100 was added to each well and incubation lasted 1 h at room temperature. When we performed 96-well plate assays using a BioTek Synergy2 Microplate Reader, 100 μL cell lysate was added to each well to detect the fluorescence intensity of DCF (excitation/emission wavelength at 485 nm/528 nm). All statistical analyses were performed with one-way ANOVA followed by Dunnett’s multiple comparisons test comparing WT with mutants using GraphPad Prism v9.3.1. DCF transport assays were repeated for at least three times.

We also conducted transport activity assay using confocal microscopy with the combination of DCF and mKate.^75^ HEK293T cells were transfected with pCAG-mKate-OATP1B1-His-Flag constructs, using PEIs in a 1:2 (w/w) ratio. The total amount of DNA per transfection was 2 μg and added in a 20-mm diameter dish with 1.0 × 10^6^ cells/mL density. Procedure of DCF uptake assay is similar to the one described above. After DCF uptake, instead of cell lysis, cells were observed under confocal microscope. OATP1B1-expressing HEK293T cells were identified by detecting mKate fluorescence using a monochromator at 543 nm excitation while the influx amount of DCF was measured by detecting DCF fluorescence using a monochromator at 488 nm excitation and the emitted light measurement ranging from 592 nm to 650 nm, 493 nm to 530 nm, respectively. Fluorescence intensity was monitored by a Leica SP8 confocal microscope. After image acquisition, quantitative image analysis was performed with ImageJ.^76^ All statistical analyses were performed with one-way ANOVA followed by Dunnett’s multiple comparisons test comparing WT with mutants using GraphPad Prism v9.3.1. DCF transport assays were repeated for at least three times.

### Cell surface biotinylation and immunoblot analysis

HEK293T cells were cultured in a 6-well plate transiently transfected with plasmids of OATP1B1 WT and mutants. After 48 h, cells were washed twice with 2 mL of ice-cold PBS and then treated for 1 h at room temperature with 1 mL of sulfo-N-hydroxysuccini- mide-SS-biotin (Thermo Fisher Scientific) (1 mg/mL in PBS). After this, cells were washed for three times with 2 mL of ice-cold PBS containing 100 mM glycine and incubated for 10 min at 4 °C in the same buffer. Then, cells were lysed with 500 μL of lysis buffer (10 mM Tris, 150 mM NaCl, 1 mM EDTA, 0.1% SDS, and 1% Triton X-100, pH 7.4, containing protease inhibitors) for 2 h at 4 °C with shaking. Lysates were centrifuged for 2 min at 10,000× *g* and the supernatants were incubated with 30 μL of streptavidin-agarose beads (Thermo Fisher Scientific) for 1 h at room temperature under constant agitation. The beads were then centrifuged at 500× *g* for 1 min, washed for five times with ice-cold lysis buffer, and incubated with 100 μL of 2× Laemmli buffer containing 100 mM dithiothreitol at room temperature for 30 min to recover the cell surface proteins. Samples were separated using SDS-PAGE followed by western blot analysis. All constructs and mutant proteins were detected using anti-Flag-HRP antibody (GNI) (1: 5000 dilution). Then the same membrane was stripped for 1 h at room temperature. Na^+^/K^+^-ATPase was used as loading control for normalization and was detected with rabbit anti-Na^+^/K^+^-ATPase monoclonal antibody (1:10,000 dilution) (ABclonal) combined with secondary HRP goat anti-rabbit monoclonal antibody (1:10,000 dilution) (ABclonal).

### Inhibition of OATP1B1-mediated uptake of DCF by compounds of B/S/E

Cells cultured in a 24-well plate were used for transport measurement. To each well, HEK293T cells were transfected with pCAG-OATP1B1-His-Flag constructs, using Lipofectamine 2000 reagent (Invitrogen) following the manufacturer’s instructions and the total amount of DNA per transfection was 0.6 μg. After 48 h, DMEM medium in the dish was aspirated and cells were washed with PBS for three times. 2.5 μM DCF in uptake buffer was mixed with each of the three compounds in six concentrations of 0.001–100 μM for E3S, simeprevir and 0.005–500 μM for bilirubin, in the total volume of 200 μL. The mixtures were then added to each well, and HEK293T cells with addition of 2.5 μM DCF in uptake buffer without compounds were used as a positive control, assumed to possess 100% activity. HEK293T cells transfected by PBS with addition of 2.5 μM DCF in uptake buffer without compounds were used as a blank control. After incubation for 5 min at 37 °C, uptake buffer with DCF and compounds was aspirated out and cells were washed with pre-cooling uptake buffer for three times. Then, 200 μL uptake buffer with 2% Triton X-100 was added to each well and incubation lasted for 1 h at room temperature. When performing 96-well plate assays using a BioTek Synergy2 Microplate Reader, 100 μL cell lysate was added to each well to detect the fluorescence intensity of DCF (excitation/emission wavelength at 485 nm/528 nm). Six experimental repeats were performed for each concentration, except that 5 experimental repeats were performed for WT in inhibition assay of bilirubin.

The fluorescence intensity of blank control was first deducted from experimental group and positive control. Then the measurements of experimental group were normalized according to positive control for final data analysis performed with nonlinear regression (curve fit) using GraphPad Prism v9.3.1.

### [^3^H]-E3S liposome counterflow assay

*Escherichia coli* polar lipid extract (Avanti) supplemented with 20% (w%) cholesterol was resuspended to 20 mg/mL in the buffer (20 mM HEPES pH 7.4, 150 mM NaCl) plus 0.2 mM estrone sulfate sodium (MedChemExpress). After 10 cycles of freeze-and-thaw by liquid nitrogen, the liposomes were extruded through 0.4 μm polycarbonate track-etched membranes (GE Healthcare) for 21 times. The liposomes were pre-incubated with 1% n-octyl-β-D-glucoside (β-OG) (Anatrace) for 30 min at 4 °C, then the liposomes were incubated with 200 μg/mL OATP1B1 (WT or mutants) for 1 h at 4 °C. Detergents were removed by incubation overnight with 250 mg/mL Bio-Beads SM2 (Bio-Rad) at 4 °C, and then the supernatant was incubated with 100 mg/mL Bio-Beads SM2 for an additional 1 h. After 5 cycles of freeze-and-thaw and extrusion for 21 times again, the proteoliposomes were collected by ultracentrifugation at 100,000× *g* for 1 h at 4 °C, washed twice and finally resuspended to 100 mg/mL with buffer for the following counterflow assay.

Each counterflow assay was performed by adding 2 μL proteoliposomes into 97 μL buffer (20 mM HEPES pH 7.4, 150 mM NaCl) and 1 μL Ammonium Salt-[6,7-^3^H(N)]-Estrone Sulfate (PerkinElmer). Uptake of radio-labeled substrates was terminated at 60 s by rapidly filtering the solution through glass fiber filters (Advantec) and washed with 3 mL ice-cold buffer. The filters were incubated with 0.5 mL Optiphase HISAFE 3 (PerkinElmer) overnight for liquid scintillation counting by MicroBeta^2®^ Microplate Counter (PerkinElmer). All experiments were repeated three times at 25 °C and all statistical analyses were performed with one-way ANOVA followed by Dunnett’s multiple comparisons test comparing WT with mutants using GraphPad Prism v9.3.1. Error bars represent SEM.

### Supplementary information

Supplementary information, Video S1

Supplementary information, Video S2

Supplementary information, Video S3

Supplementary information, Video S4

Supplementary information, Video S5

Supplementary information, Table S1

Supplementary information, Table S2

Supplementary video S1 legend

Supplementary video S2 legend

Supplementary video S3 legend

Supplementary video S4 legend

Supplementary video S5 legend

Supplementary information, Fig. S1

Supplementary information, Fig. S2

Supplementary information, Fig. S3

Supplementary information, Fig. S4

Supplementary information, Fig. S5

Supplementary information, Fig. S6

Supplementary information, Fig. S7

Supplementary information, Fig. S8

Supplementary information, Fig. S9

Supplementary information, Fig. S10

Supplementary information, Fig. S11

Supplementary information, Fig. S12

Supplementary information, Fig. S13

Supplementary information, Fig. S14

Supplementary information, Fig. S15

## Data Availability

Atomic coordinates of OATP1B1-apo/B/S/E_in_/D have been deposited in the Protein Data Bank (http://www.rcsb.org) under the accession codes 8HNB, 8HNC, 8HNH, 8HND, and 8K6L, respectively. The corresponding electron microscopy maps have been deposited in the Electron Microscopy Data Bank (https://www.ebi.ac.uk/pdbe/emdb/), under the accession codes EMD-34909, EMD-34910, EMD-34913, EMD-34911, and EMD-36922, respectively.
