# Supplementary material for: Cryo-EM structures of human organic anion transporting polypeptide OATP1B1
Source: Cell Res. 2023 Sep 6;33(12):940–51. doi: 10.1038/s41422-023-00870-8 (PMC10709409; doi:10.1038/s41422-023-00870-8)
Supplement: Supplementary file 6 — Supplementary information, Table S1 [file 41422_2023_870_MOESM6_ESM.pdf]

Supplementary information, Table S1 Cryo-EM data collection, refinement and validation statistics.

	OATP1B1-*apo*	OATP1B1-B	OATP1B1-S	OATP1B1-E_{in}	OATP1B1-D
EMDB	(34909)	(34910)	(34913)	(34911)	(36922)
PDB	(8HNB)	(8HNC)	(8HNH)	(8HND)	(8K6L)
**Data collection and processing**					
Magnification	105,000	105,000	105,000	130,000	130,000
Voltage (kV)	300	300	300	300	300
Electron exposure ($e^{-}/Å^2$)	49.87	49.24	50.21	49.32	46.10
Defocus range (μm)	-1.0 to -1.8	-1.0 to -1.8	-1.0 to -1.8	-0.9 to -1.5	-0.9 to -1.5
Pixel size (Å)	0.832	0.832	0.832	0.932	0.932
Symmetry imposed	C1	C1	C1	C1	C1
Initial particle images (no.)	15,917,707	19,107,481	10,323,938	11,779,119	6,992,221
Final particle images (no.)	325,578	77,213	149,222	105,261	325,151
Map resolution (Å)	3.53	3.73	3.73	3.19	2.92
FSC threshold	0.143	0.143	0.143	0.143	0.143
**Refinement**					
Initial model used (PDB code)	SWISS-MODEL	8HNB	8HNB	AlphaFold	8HNB
Map sharpening *B* factor ($Å^2$)	-100.0	-196.3	-208.5	-159.3	-126.9
Model composition					
Non-hydrogen atoms	4,084	4,273	4,237	4,370	4,156
Protein residues	562	570	563	560	547
Ligands	N/A	Bilirubin	Simeprevir	E3S	DCF
B factors ($Å^2$)					
Protein	66.37	63.44	37.63	91.30	57.84
Ligand	N/A	83.17	153.36	111.14	80.33
R.m.s. deviations					
Bond lengths (Å)	0.006	0.010	0.01	0.007	0.005
Bond angles (°)	1.384	1.303	1.538	1.250	1.134
Validation					
MolProbity score	1.96	1.93	2.34	1.96	1.99
Clashscore	13.49	14.34	16.98	13.77	6.95
Poor rotamers (%)	2.09	0.24	0.00	0.65	2.17
Ramachandran plot					
Favored (%)	97.65	96.09	96.94	95.47	94.99
Allowed (%)	2.35	3.91	3.06	4.53	5.01
Disallowed (%)	0.00	0.00	0.00	0.00	0.00
