# Supplementary material for: Cryo-EM structures of human organic anion transporting polypeptide OATP1B1
Source: Cell Res. 2023 Sep 6;33(12):940–51. doi: 10.1038/s41422-023-00870-8 (PMC10709409; doi:10.1038/s41422-023-00870-8)
Supplement: Supplementary file 7 — Supplementary information, Table S2 [file 41422_2023_870_MOESM7_ESM.pdf]

Supplementary information, Table S2 Systems in MD simulations

Systems	Protein conformtion	Substrate	No. of POPC	No. of Water	No.of Na^+	No.of Cl^-	Total atoms	Replicas	Simulation time (ns)
OATP1B1-*apo*	Outward	N/A	349	42512	187	205	184529	3	500
OATP1B1-B	Outward	Bilirubin	350	42417	187	203	184453	3	500
OATP1B1-S	Outward	Simeprevir	351	42466	187	205	184758	3	500
OATP1B1-E_{in}	Inward	Estrone-3-sulfate	356	42387	187	207	185143	3	500
OATP1B1-D	Outward	DCF	356	42575	187	205	185693	3	500
