# Supplementary material for: Cryo-EM structures of human organic anion transporting polypeptide OATP1B1
Source: Cell Res. 2023 Sep 6;33(12):940–51. doi: 10.1038/s41422-023-00870-8 (PMC10709409; doi:10.1038/s41422-023-00870-8)
Supplement: Supplementary file 10 — Supplementary video S3 legend [file 41422_2023_870_MOESM10_ESM.pdf]

Supplementary information, Video S3 MD simulations of OATP1B1-E_{in}. A representative movie of a 500-*ns* MD simulation of OATP1B1-E_{in}. E3S (green) and functionally crucial residues Y352 (cyan), F356 (blue) and F386 (purple) are shown as sticks. OATP1B1 in the cartoon is colored by its NTD (gold) and CTD (marine).
