# Supplementary material for: Cryo-EM structures of human organic anion transporting polypeptide OATP1B1
Source: Cell Res. 2023 Sep 6;33(12):940–51. doi: 10.1038/s41422-023-00870-8 (PMC10709409; doi:10.1038/s41422-023-00870-8)
Supplement: Supplementary file 12 — Supplementary video S5 legend [file 41422_2023_870_MOESM12_ESM.pdf]

Supplementary information, Video S5 Rocker-switch model of OATP1B1 during transport. Morph between outward-open structure of OATP1B1-*apo* (initial state), and inward-open structure of OATP1B1-E_{in}. NTD and CTD are colored with gold and marine, respectively. Morph was generated by PyMOL.
