# Supplementary material for: Cryo-EM structures of human organic anion transporting polypeptide OATP1B1
Source: Cell Res. 2023 Sep 6;33(12):940–51. doi: 10.1038/s41422-023-00870-8 (PMC10709409; doi:10.1038/s41422-023-00870-8)
Supplement: Supplementary file 13 — Supplementary information, Fig. S1 [file 41422_2023_870_MOESM13_ESM.pdf]

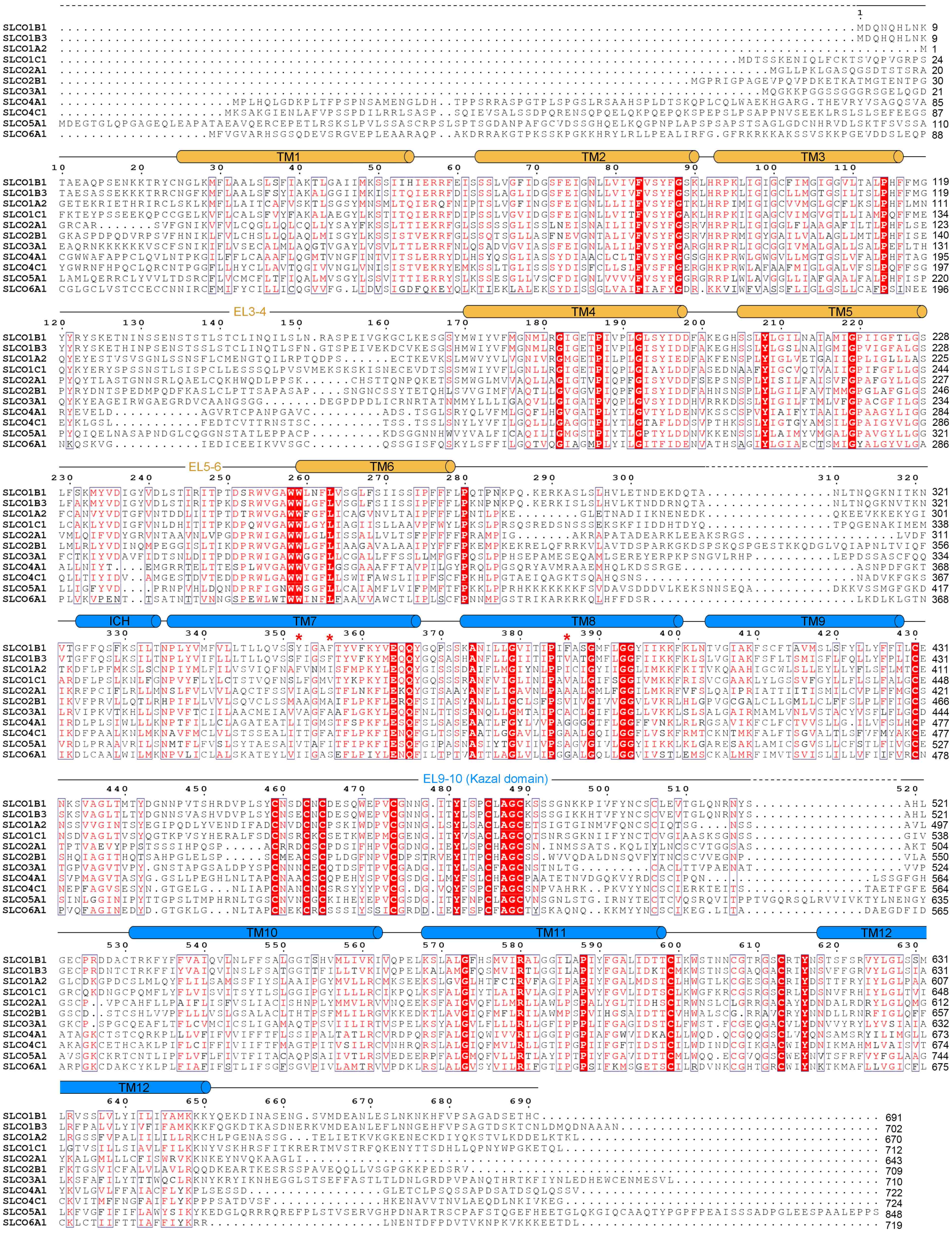

SLCO1B1
SLCO1B3
SLCO1A2
SLCO1C1
SLCO2A1
SLCO2B1
SLCO3A1
SLCO4A1
SLCO4C1
SLCO5A1
SLCO6A1
TM1
TM2
TM3
EL3-4
TM4
TM5
EL5-6
TM6
ICH
TM7
TM8
TM9
EL9-10 (Kazal domain)
TM10
TM11
TM12

Supplementary information, Fig. S1 Sequence alignment of SLCO family transporters. Sequence alignment among human SLCO family transporters. The UniProt IDs for the aligned sequences are: SLCO1B1: Q9Y6L6; SLCO1B3: Q9NPD5; SCLO1A2: P46721; SCLO1C1: Q9NYB5; SLCO2A1: Q92959; SLCO2B1: O94956; SLCO3A1: Q9UIG8; SLCO4A1: Q96BD0; SLCO4C1: Q6ZQN7; SLCO5A1: Q9H2Y9; SLCO6A1: Q86UG4. TMs and ELs are labeled, with NTD and CTD colored in gold and marine, respectively. Crucial residues involved in substrate binding in central cavity, Y352, F356 and F386, are labeled as red asterisk.
