# Supplementary material for: Cryo-EM structures of human organic anion transporting polypeptide OATP1B1
Source: Cell Res. 2023 Sep 6;33(12):940–51. doi: 10.1038/s41422-023-00870-8 (PMC10709409; doi:10.1038/s41422-023-00870-8)
Supplement: Supplementary file 14 — Supplementary information, Fig. S2 [file 41422_2023_870_MOESM14_ESM.pdf]

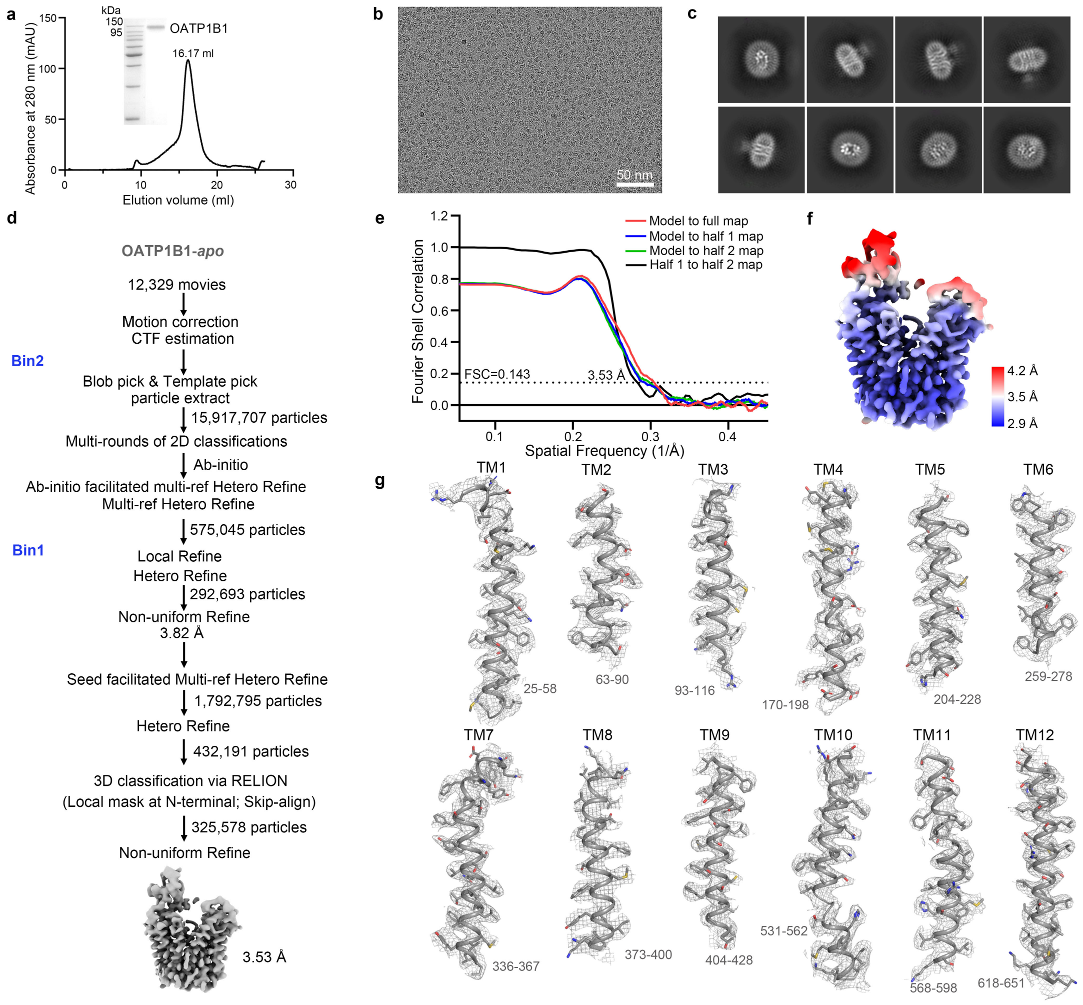
a
kDa
150
95
OATP1B1
16.17 ml
Absorbance at 280 nm (mAU)
150
100
50
0
0
10
20
30
Elution volume (ml)
b
50 nm
c
d
OATP1B1-apo
12,329 movies
Motion correction
CTF estimation
Bin2
Blob pick & Template pick
particle extract
15,917,707 particles
Multi-rounds of 2D classifications
Ab-initio
Ab-initio facilitated multi-ref Hetero Refine
Multi-ref Hetero Refine
575,045 particles
Bin1
Local Refine
Hetero Refine
292,693 particles
Non-uniform Refine
3.82 Å
Seed facilitated Multi-ref Hetero Refine
1,792,795 particles
Hetero Refine
432,191 particles
3D classification via RELION
(Local mask at N-terminal; Skip-align)
325,578 particles
Non-uniform Refine
3.53 Å
e
Model to full map
Model to half 1 map
Model to half 2 map
Half 1 to half 2 map
Fourier Shell Correlation
1.2
1.0
0.8
0.6
0.4
0.2
0.0
FSC=0.143
3.53 Å
0.1
0.2
0.3
0.4
Spatial Frequency (1/Å)
f
4.2 Å
3.5 Å
2.9 Å
g
TM1
TM2
TM3
TM4
TM5
TM6
25-58
63-90
93-116
170-198
204-228
259-278
TM7
TM8
TM9
TM10
TM11
TM12
336-367
373-400
404-428
531-562
568-598
618-651

Supplementary information, Fig. S2 Cryo-EM data processing for OATP1B1 in *apo* state. **a** Size-exclusion chromatography of OATP1B1 by Superose 6 (GE Healthcare) and the protein peak detected by SDS-PAGE gel. **b** A representative cryo-EM micrograph. Scale bar: 50 nm. **c** Representative 2D class averages generated from particles for OATP1B1-*apo* map. **d** Flowchart of cryo-EM data processing of OATP1B1-*apo*. **e** Gold-standard FSC curve between two half maps (black) with indicated resolution at 0.143, and FSC curves between the atomic model refined against full map (red) and half 1 map (blue) or half 2 map (green). **f** The local resolution map of OATP1B1-*apo*. **g** The cryo-EM density maps for all transmembrane helices shown as mesh (5σ) colored in gray, with atomic models shown as cartoon and sidechains shown as sticks, and colored in gray.
