# Supplementary material for: Cryo-EM structures of human organic anion transporting polypeptide OATP1B1
Source: Cell Res. 2023 Sep 6;33(12):940–51. doi: 10.1038/s41422-023-00870-8 (PMC10709409; doi:10.1038/s41422-023-00870-8)
Supplement: Supplementary file 15 — Supplementary information, Fig. S3 [file 41422_2023_870_MOESM15_ESM.pdf]

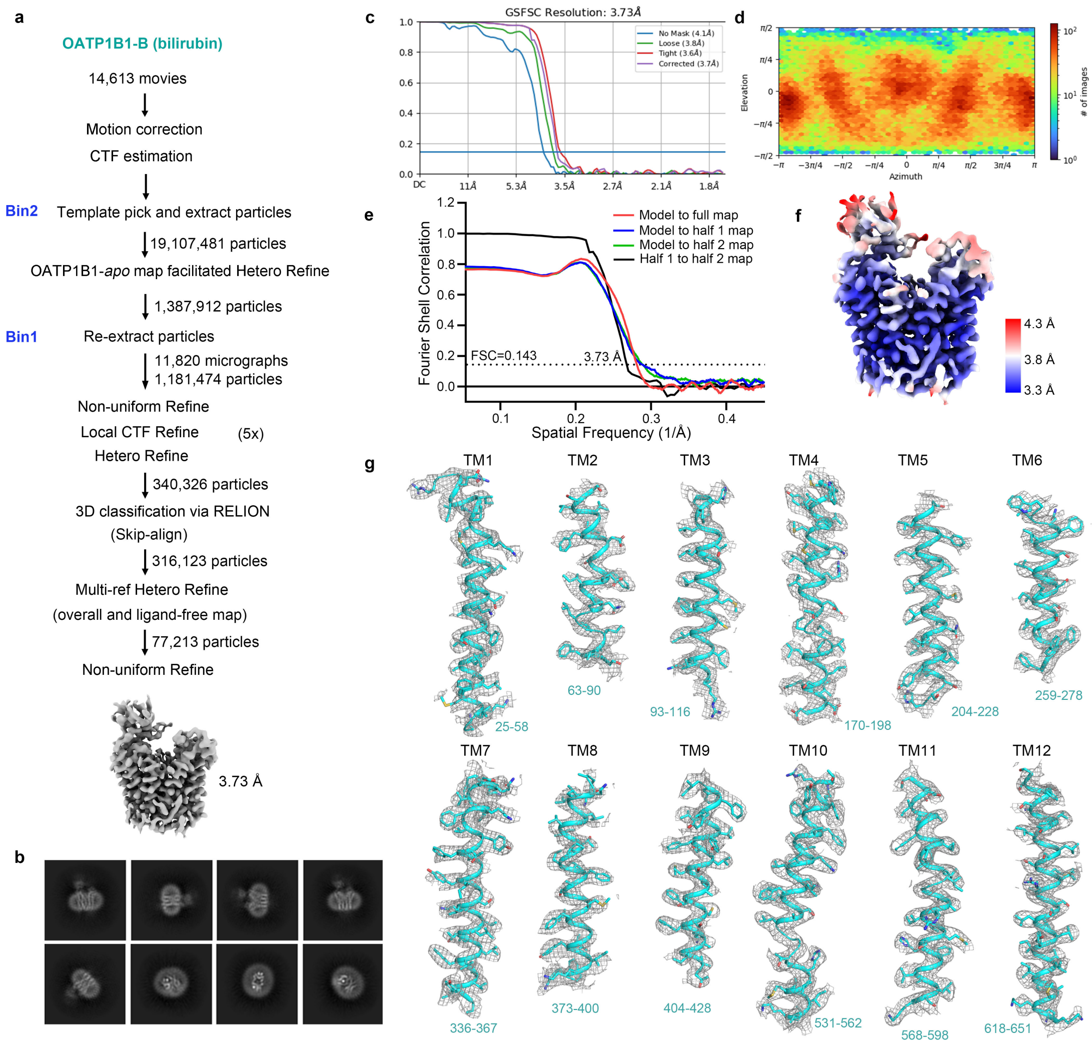

a
OATP1B1-B (bilirubin)
14,613 movies
Motion correction
CTF estimation
Bin2
Template pick and extract particles
19,107,481 particles
OATP1B1-apo map facilitated Hetero Refine
1,387,912 particles
Bin1
Re-extract particles
11,820 micrographs
1,181,474 particles
Non-uniform Refine
Local CTF Refine
(5x)
Hetero Refine
340,326 particles
3D classification via RELION
(Skip-align)
316,123 particles
Multi-ref Hetero Refine
(overall and ligand-free map)
77,213 particles
Non-uniform Refine
3.73 Å
b
c
GSFSC Resolution: 3.73Å
No Mask (4.1Å)
Loose (3.8Å)
Tight (3.6Å)
Corrected (3.7Å)
DC
11Å
5.3Å
3.5Å
2.7Å
2.1Å
1.8Å
d
Elevation
Azimuth
# of images
e
Fourier Shell Correlation
Spatial Frequency (1/Å)
Model to full map
Model to half 1 map
Model to half 2 map
Half 1 to half 2 map
FSC=0.143
3.73 Å
f
4.3 Å
3.8 Å
3.3 Å
g
TM1
TM2
TM3
TM4
TM5
TM6
25-58
63-90
93-116
170-198
204-228
259-278
TM7
TM8
TM9
TM10
TM11
TM12
336-367
373-400
404-428
531-562
568-598
618-651

Supplementary information, Fig. S3 Cryo-EM data processing for OATP1B1 bound to bilirubin. a Flowchart of cryo-EM data processing of OATP1B1-B. **b** Representative 2D class averages generated from particles for OATP1B1-B map. **c** Fourier Shell Correlation (FSC) curves generated by cryoSPARC. **d** Angular distributions for particles contributing to the cryo-EM map of OATP1B1-B. **e** Gold-standard FSC curve between two half maps (black) with indicated resolution at 0.143, and FSC curves between the atomic model refined against full map (red) and half 1 map (blue) or half 2 map (green). **f** The local resolution map of OATP1B1-B. **g** The cryo-EM density maps for all transmembrane helices shown as mesh (5σ) colored in gray, with atomic models shown as cartoon and sidechains shown as sticks, and colored in cyan.
