# Supplementary material for: Cryo-EM structures of human organic anion transporting polypeptide OATP1B1
Source: Cell Res. 2023 Sep 6;33(12):940–51. doi: 10.1038/s41422-023-00870-8 (PMC10709409; doi:10.1038/s41422-023-00870-8)
Supplement: Supplementary file 17 — Supplementary information, Fig. S5 [file 41422_2023_870_MOESM17_ESM.pdf]

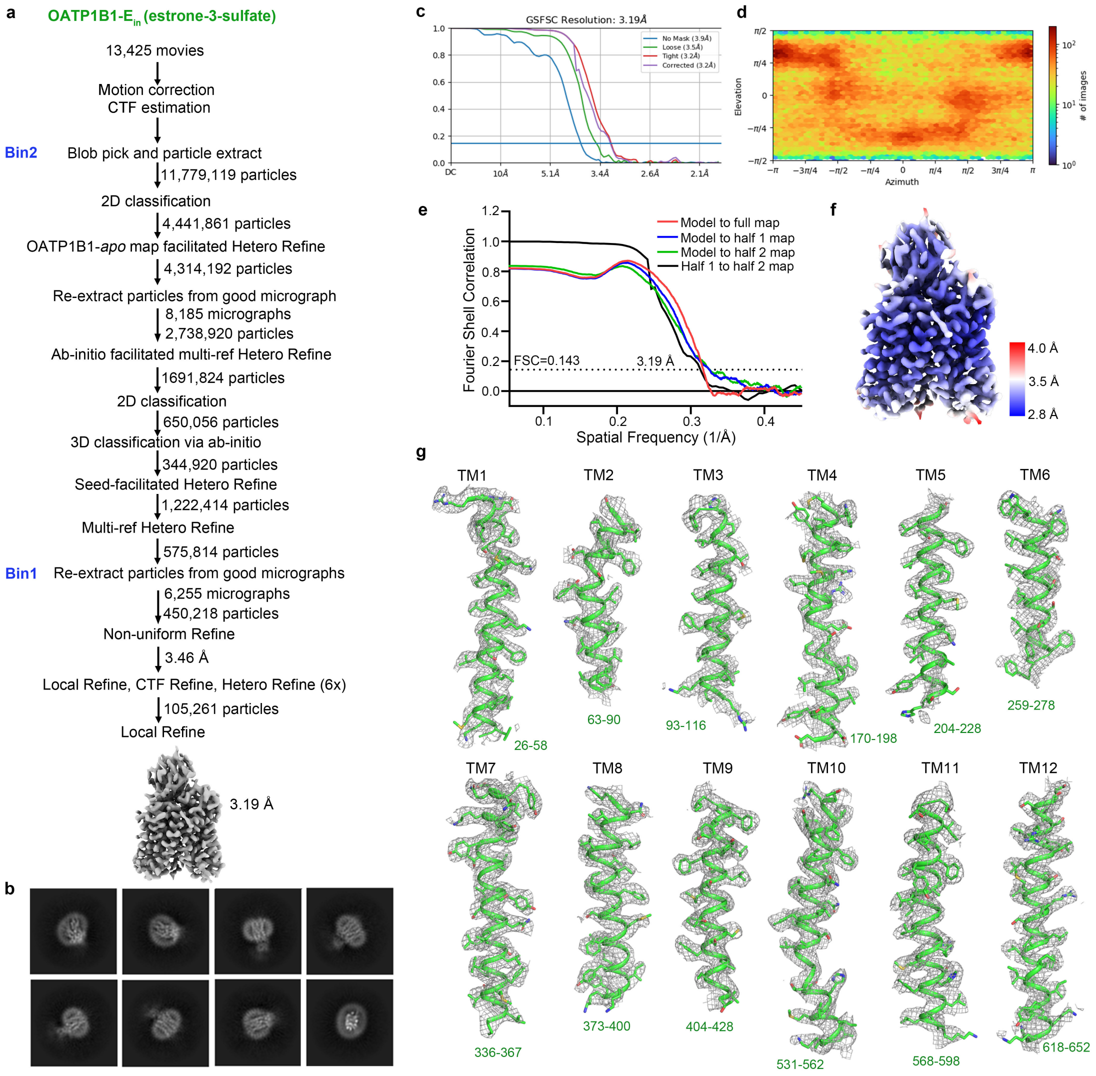
a
OATP1B1-E$_{in}$ (estrone-3-sulfate)
13,425 movies
Motion correction
CTF estimation
Bin2
Blob pick and particle extract
11,779,119 particles
2D classification
4,441,861 particles
OATP1B1-*apo* map facilitated Hetero Refine
4,314,192 particles
Re-extract particles from good micrograph
8,185 micrographs
2,738,920 particles
Ab-initio facilitated multi-ref Hetero Refine
1691,824 particles
2D classification
650,056 particles
3D classification via ab-initio
344,920 particles
Seed-facilitated Hetero Refine
1,222,414 particles
Multi-ref Hetero Refine
575,814 particles
Bin1
Re-extract particles from good micrographs
6,255 micrographs
450,218 particles
Non-uniform Refine
3.46 Å
Local Refine, CTF Refine, Hetero Refine (6x)
105,261 particles
Local Refine
3.19 Å
b
c
GSFSC Resolution: 3.19Å
No Mask (3.9Å)
Loose (3.5Å)
Tight (3.2Å)
Corrected (3.2Å)
d
Elevation
Azimuth
# of images
e
Fourier Shell Correlation
Spatial Frequency (1/Å)
Model to full map
Model to half 1 map
Model to half 2 map
Half 1 to half 2 map
FSC=0.143
3.19 Å
f
4.0 Å
3.5 Å
2.8 Å
g
TM1
26-58
TM2
63-90
TM3
93-116
TM4
170-198
TM5
204-228
TM6
259-278
TM7
336-367
TM8
373-400
TM9
404-428
TM10
531-562
TM11
568-598
TM12
618-652

Supplementary information, Fig. S5 Cryo-EM data processing for OATP1B1 bound to E3S. **a** Flowchart of cryo-EM data processing of OATP1B1-E_{in}. **b** Representative 2D class averages generated from particles for OATP1B1-E_{in} map. **c** Fourier Shell Correlation (FSC) curves generated by cryoSPARC. **d** Angular distributions for particles contributing to the cryo-EM map of OATP1B1-E_{in}. **e** Gold-standard FSC curve between two half maps (black) with indicated resolution at 0.143, and FSC curves between the atomic model refined against full map (red) and half 1 map (blue) or half 2 map (green). **f** The local resolution map of OATP1B1-E_{in}. **g** The cryo-EM density maps for all transmembrane helices shown as mesh (5σ) colored in gray, with atomic models shown as cartoon and sidechains shown as sticks, and colored in green.
