# Supplementary material for: Cryo-EM structures of human organic anion transporting polypeptide OATP1B1
Source: Cell Res. 2023 Sep 6;33(12):940–51. doi: 10.1038/s41422-023-00870-8 (PMC10709409; doi:10.1038/s41422-023-00870-8)
Supplement: Supplementary file 18 — Supplementary information, Fig. S6 [file 41422_2023_870_MOESM18_ESM.pdf]

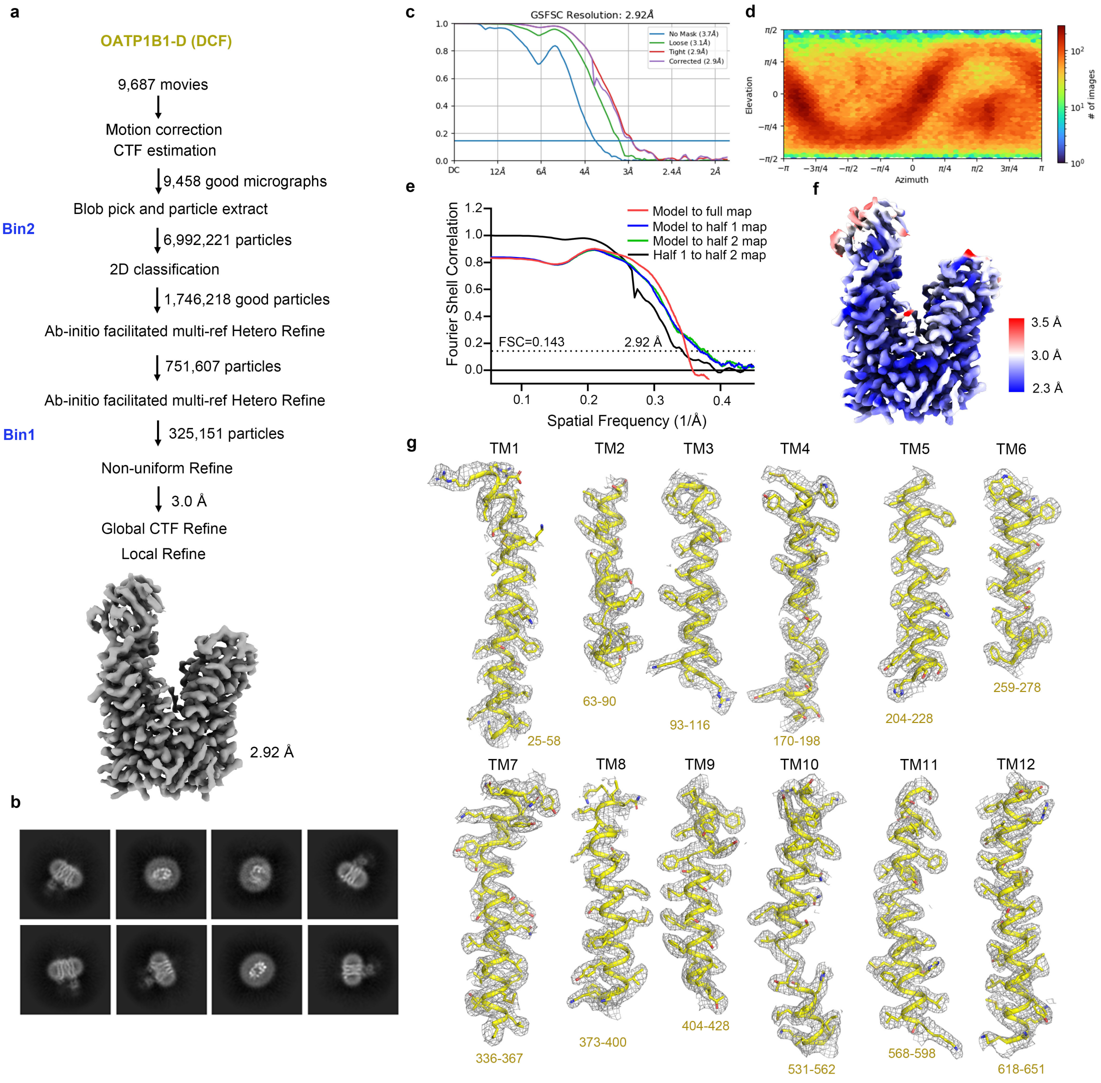
a
OATP1B1-D (DCF)
9,687 movies
Motion correction
CTF estimation
9,458 good micrographs
Blob pick and particle extract
Bin2
6,992,221 particles
2D classification
1,746,218 good particles
Ab-initio facilitated multi-ref Hetero Refine
751,607 particles
Ab-initio facilitated multi-ref Hetero Refine
Bin1
325,151 particles
Non-uniform Refine
3.0 Å
Global CTF Refine
Local Refine
2.92 Å
b
c
GSFSC Resolution: 2.92Å
No Mask (3.7Å)
Loose (3.1Å)
Tight (2.9Å)
Corrected (2.9Å)
DC
12Å
6Å
4Å
3Å
2.4Å
2Å
d
Elevation
Azimuth
# of images
e
Fourier Shell Correlation
Spatial Frequency (1/Å)
Model to full map
Model to half 1 map
Model to half 2 map
Half 1 to half 2 map
FSC=0.143
2.92 Å
f
3.5 Å
3.0 Å
2.3 Å
g
TM1
TM2
TM3
TM4
TM5
TM6
25-58
63-90
93-116
170-198
204-228
259-278
TM7
TM8
TM9
TM10
TM11
TM12
336-367
373-400
404-428
531-562
568-598
618-651

Supplementary information, Fig. S6 Cryo-EM data processing for OATP1B1 bound to DCF. **a** Flowchart of cryo-EM data processing of OATP1B1-D. **b** Representative 2D class averages generated from particles for OATP1B1-D map. **c** Fourier Shell Correlation (FSC) curves generated by cryoSPARC. **d** Angular distributions for particles contributing to the cryo-EM map of OATP1B1-D. **e** Gold-standard FSC curve between two half maps (black) with indicated resolution at 0.143, and FSC curves between the atomic model refined against full map (red) and half 1 map (blue) or half 2 map (green). **f** The local resolution map of OATP1B1-D. **g** The cryo-EM density maps for all transmembrane helices shown as mesh (5σ) colored in gray, with atomic models shown as cartoon and sidechains shown as sticks, and colored in yellow.
