# Supplementary material for: Cryo-EM structures of human organic anion transporting polypeptide OATP1B1
Source: Cell Res. 2023 Sep 6;33(12):940–51. doi: 10.1038/s41422-023-00870-8 (PMC10709409; doi:10.1038/s41422-023-00870-8)
Supplement: Supplementary file 19 — Supplementary information, Fig. S7 [file 41422_2023_870_MOESM19_ESM.pdf]

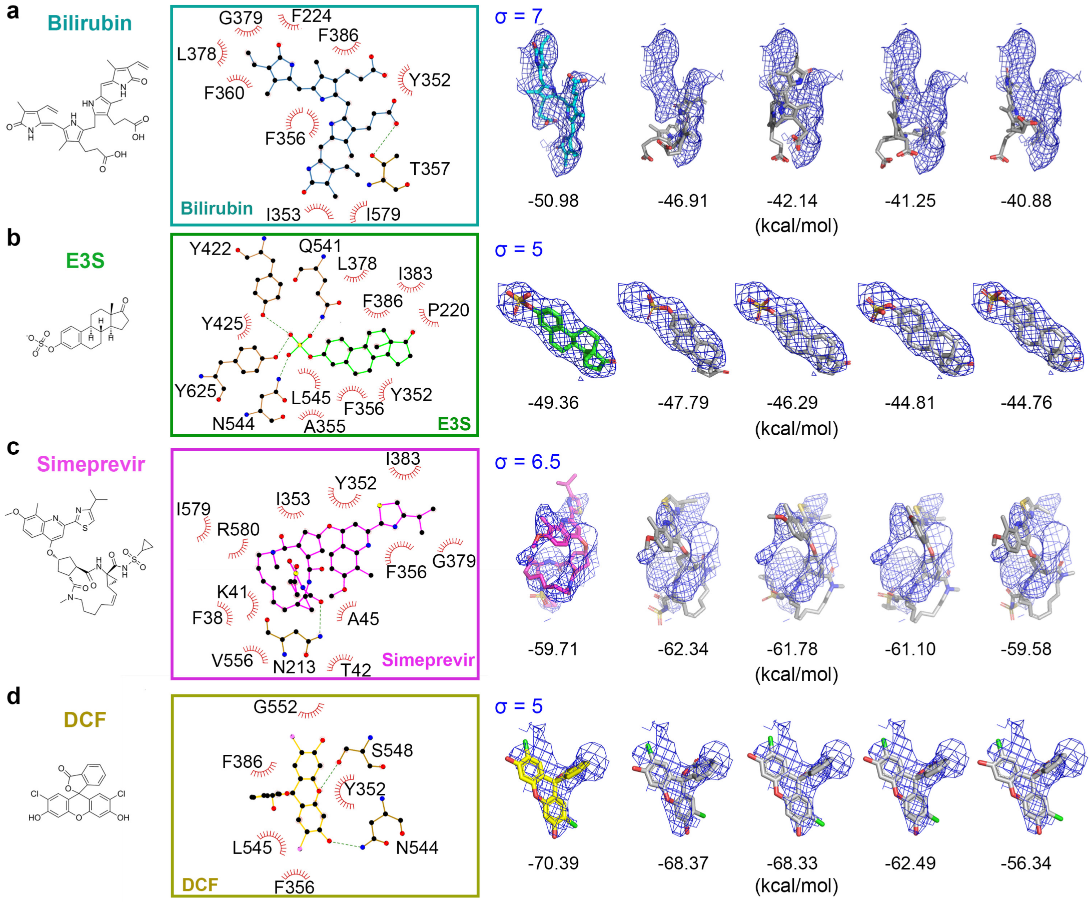
a
Bilirubin
G379
F224
F386
L378
F360
Y352
F356
T357
Bilirubin
I353
I579
σ = 7
-50.98
-46.91
-42.14
-41.25
-40.88
(kcal/mol)
b
E3S
Y422
Q541
L378
I383
F386
P220
Y425
Y625
L545
F356
Y352
N544
A355
E3S
σ = 5
-49.36
-47.79
-46.29
-44.81
-44.76
(kcal/mol)
c
Simeprevir
I383
Y352
I353
I579
R580
F356
G379
K41
F38
A45
V556
N213
T42
Simeprevir
σ = 6.5
-59.71
-62.34
-61.78
-61.10
-59.58
(kcal/mol)
d
DCF
G552
S548
F386
Y352
L545
N544
DCF
F356
σ = 5
-70.39
-68.37
-68.33
-62.49
-56.34
(kcal/mol)

Supplementary information, Fig. S7 Ligand conformations and interactions with OATP1B1. a-d Chemical structure (left panel), LigPlot^{+} analysis (middle panel) and EM density and cartoon representation of ligand (right panel) for bilirubin (**a**), E3S (**b**), simeprevir (**c**) and DCF (**d**), respectively. In the right panel, ligand conformations that are adopted in this study is colored in cyan, green, magenta and yellow, respectively, for the four ligands. Top 4 poses of ligands resulted from free docking are shown in gray, ranked by MM/GBSA dG binding energy (kcal/mol). The contour level for the electron density map of each ligand is indicated.
