# Supplementary material for: Cryo-EM structures of human organic anion transporting polypeptide OATP1B1
Source: Cell Res. 2023 Sep 6;33(12):940–51. doi: 10.1038/s41422-023-00870-8 (PMC10709409; doi:10.1038/s41422-023-00870-8)
Supplement: Supplementary file 20 — Supplementary information, Fig. S8 [file 41422_2023_870_MOESM20_ESM.pdf]

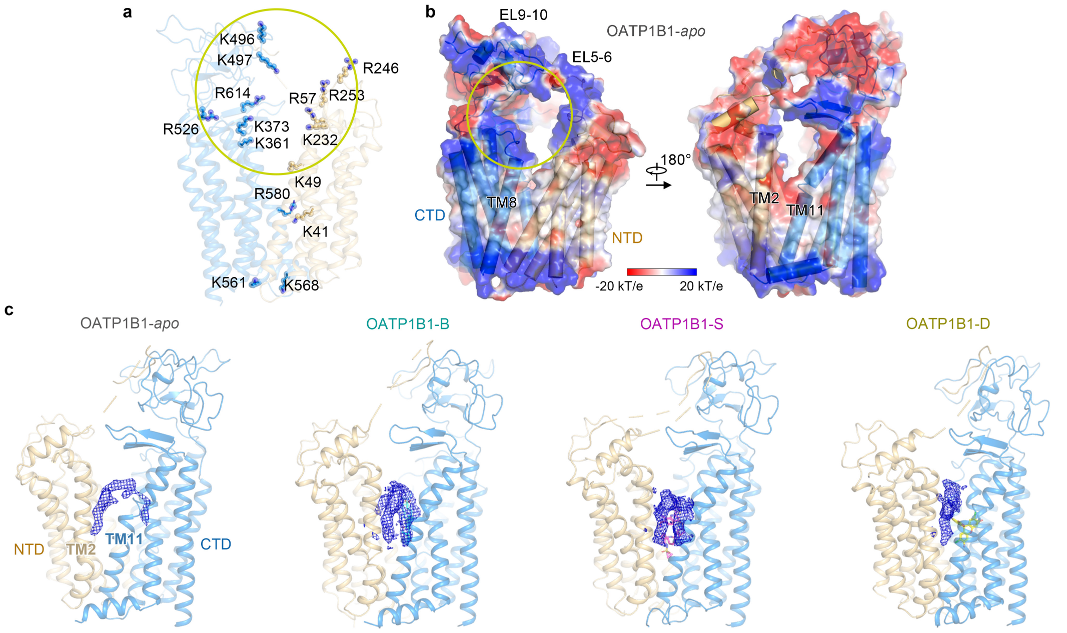

Supplementary information, Fig. S8 Cavity entrance and lateral access of substrate transport. a Electropositive residues along the translocation path of outward-open OATP1B1-*apo* and residues at the extracellular entrance are circled. **b** The surface electrostatic potential shows an electropositive opening formed mainly by EL9-10, EL5-6 and N-termini of TM8 in outward-open structure. **c** Cartoon representation of OATP1B1-*apo*/B/S/D with lipid-like densities shown in mesh.
