# Supplementary material for: Cryo-EM structures of human organic anion transporting polypeptide OATP1B1
Source: Cell Res. 2023 Sep 6;33(12):940–51. doi: 10.1038/s41422-023-00870-8 (PMC10709409; doi:10.1038/s41422-023-00870-8)
Supplement: Supplementary file 21 — Supplementary information, Fig. S9 [file 41422_2023_870_MOESM21_ESM.pdf]

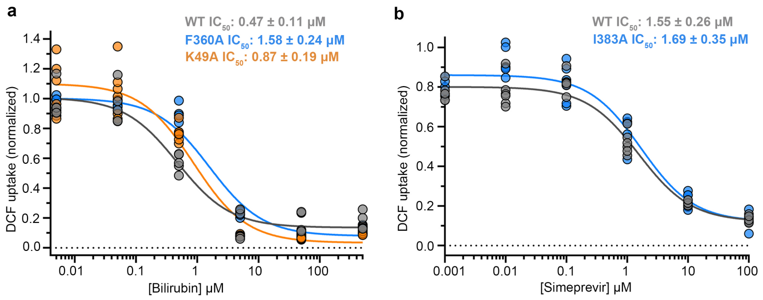


**Supplementary information, Fig. S9 Inhibition of OATP1B1 transport by bilirubin and simeprevir.** Inhibition of OATP1B1 transport by bilirubin (**a**) and simeprevir (**b**) (mean ± SEM, $n$ = 6). $n$ = 6 represent six biologically independent experiments for each cell line.
