# Supplementary material for: Cryo-EM structures of human organic anion transporting polypeptide OATP1B1
Source: Cell Res. 2023 Sep 6;33(12):940–51. doi: 10.1038/s41422-023-00870-8 (PMC10709409; doi:10.1038/s41422-023-00870-8)
Supplement: Supplementary file 22 — Supplementary information, Fig. S10 [file 41422_2023_870_MOESM22_ESM.pdf]

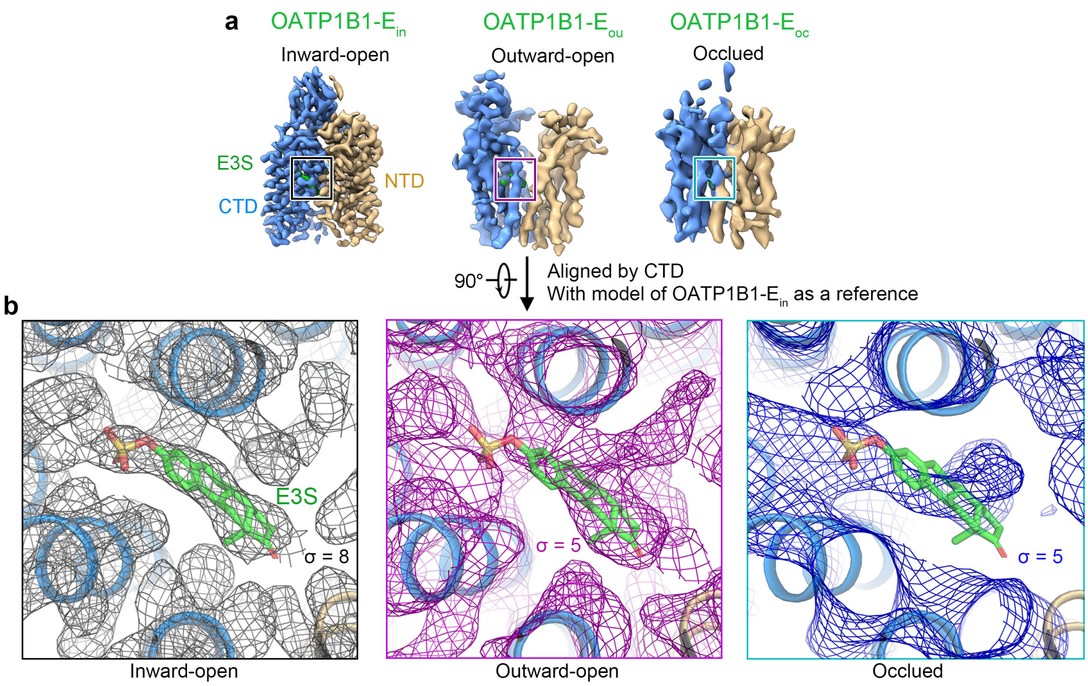

Supplementary information, Fig. S10 Ligand densities in the three conformations of OATP1B1 bound to E3S. a Cryo-EM maps of OATP1B1-E_{in}, OATP1B1-E_{ou} and OATP1B1-E_{oc}. **b** Local ligand density representations of E3S in OATP1B1-E_{in} and possible densities of E3S in OATP1B1-E_{ou} and OATP1B1-E_{oc}. All maps are aligned by CTD with model of OATP1B1-E_{in} shown as cartoon as a reference.
