# Supplementary material for: Cryo-EM structures of human organic anion transporting polypeptide OATP1B1
Source: Cell Res. 2023 Sep 6;33(12):940–51. doi: 10.1038/s41422-023-00870-8 (PMC10709409; doi:10.1038/s41422-023-00870-8)
Supplement: Supplementary file 23 — Supplementary information, Fig. S11 [file 41422_2023_870_MOESM23_ESM.pdf]

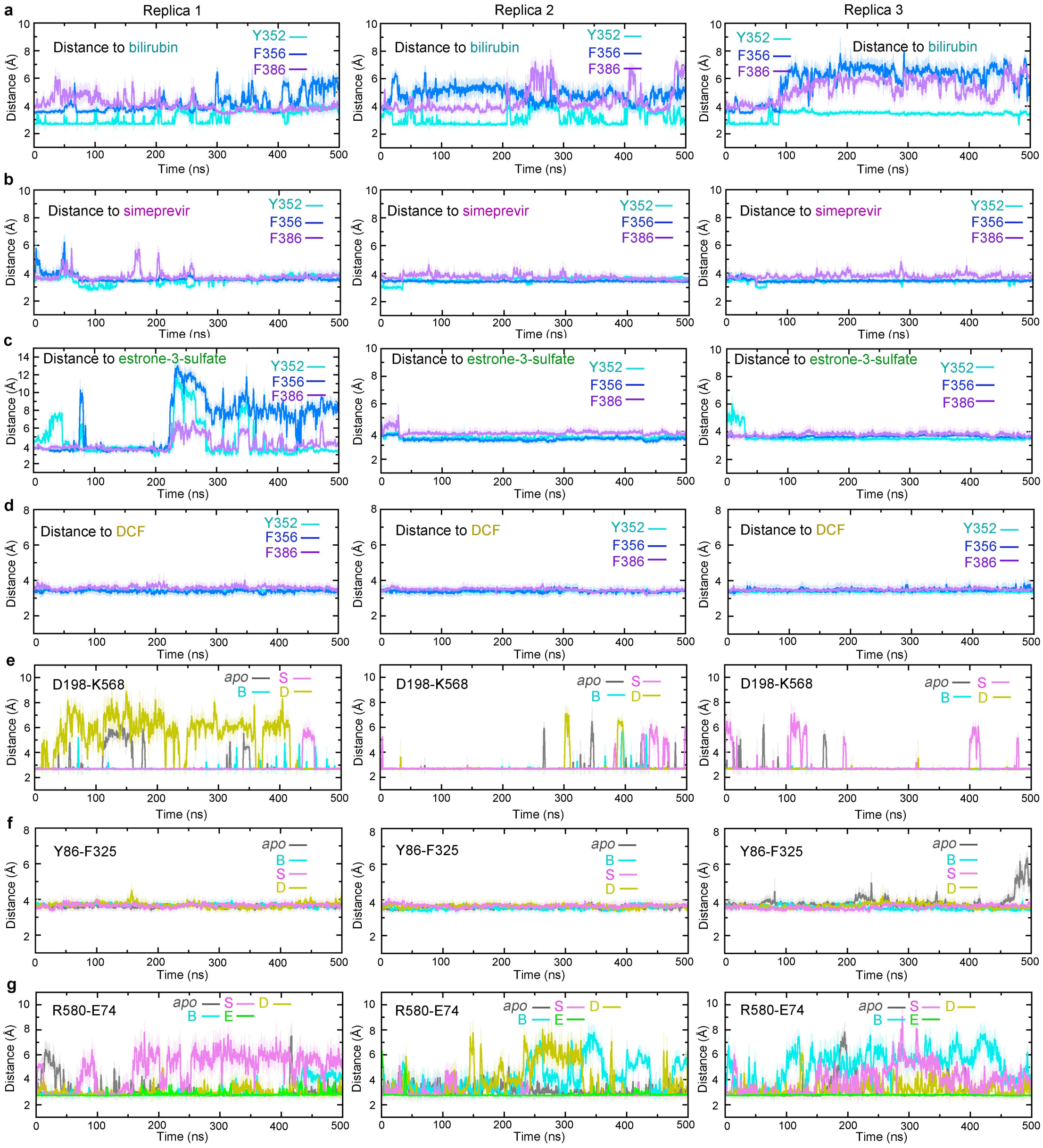
a
Replica 1
Replica 2
Replica 3
Distance to bilirubin
Y352
F356
F386
Distance (Å)
Time (ns)
b
Distance to simeprevir
c
Distance to estrone-3-sulfate
d
Distance to DCF
e
D198-K568
apo
S
B
D
f
Y86-F325
g
R580-E74
E

Supplementary information, Fig. S11 MD simulations. a-d The distances between center of mass of ligands and sidechains of Y352 (cyan), F356 (blue) and F386 (purple) for bilirubin (**a**), simeprevir (**b**) and E3S (**c**) and DCF (**d**) in three replicas. **e** The distances between center of mass of D198 sidechain carboxyl group and K568 sidechain N atom, showing the stable interaction between D198 and K568 in outward-open states. **f** The distances between center of mass of Y86 and F325 sidechain, displaying the stable attachment between Y86 and F325 in outward-open states. **g** The distances between center of mass of R580 sidechain azide group and E74 sidechain carboxyl group. MD simulations were run for OATP1B1-*apo*/B/S/E/D each for 500 ns in three replicas. *apo*, OATP1B1-*apo*; B, OATP1B1-B (bilirubin); S, OATP1B1-S (simeprevir); E, OATP1B1-E_{in} (E3S); D, OATP1B1-D (DCF).
