# Supplementary material for: Cryo-EM structures of human organic anion transporting polypeptide OATP1B1
Source: Cell Res. 2023 Sep 6;33(12):940–51. doi: 10.1038/s41422-023-00870-8 (PMC10709409; doi:10.1038/s41422-023-00870-8)
Supplement: Supplementary file 24 — Supplementary information, Fig. S12 [file 41422_2023_870_MOESM24_ESM.pdf]

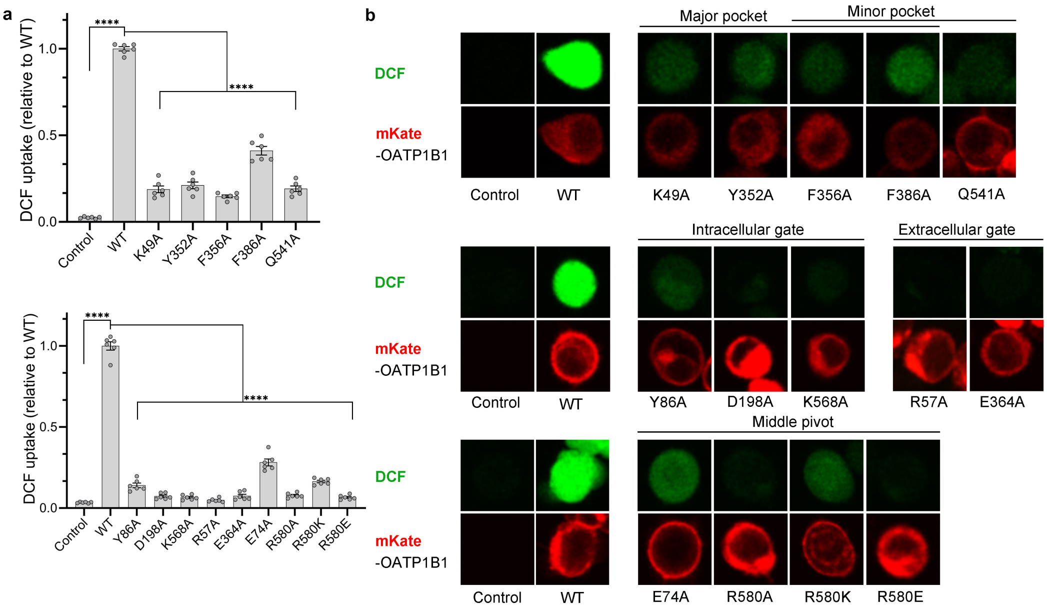

Supplementary information, Fig. S12 DCF transport activity of OATP1B1 determined by confocal microscopy. a DCF uptake activity assay of OATP1B1 mutants. The activity was normalized to the wild-type transporter (WT) (mean ± SEM, *n* = 6). Control indicates empty cell control. **b** Representative fluorescence images of cells expressing mKate-tagged OATP1B1 WT and mutants in DCF transport activity assay, captured under a Leica SP8 confocal microscope. Residue mutations are respectively related to substrate binding, intra and extracellular gate and middle pivot.
