# Supplementary material for: Cryo-EM structures of human organic anion transporting polypeptide OATP1B1
Source: Cell Res. 2023 Sep 6;33(12):940–51. doi: 10.1038/s41422-023-00870-8 (PMC10709409; doi:10.1038/s41422-023-00870-8)
Supplement: Supplementary file 25 — Supplementary information, Fig. S13 [file 41422_2023_870_MOESM25_ESM.pdf]

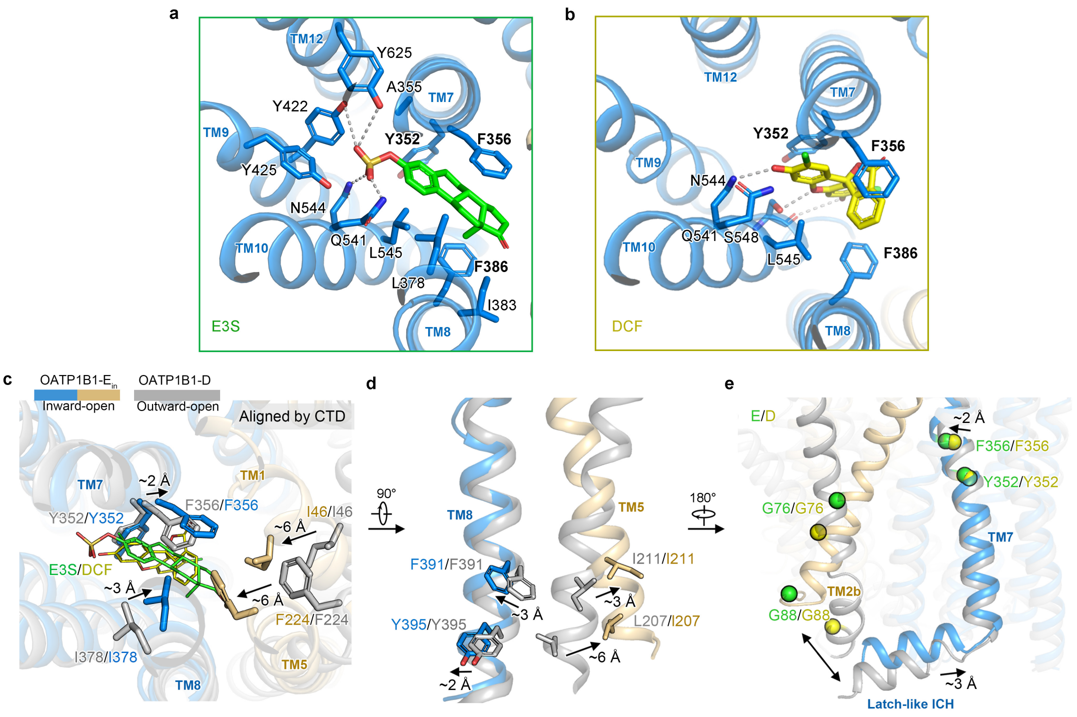

Supplementary information, Fig. S13 Structural comparison between OATP1B1-D and OATP1B1-E$_{in}$. a Close-up view of E3S binding site in OATP1B1-E$_{in}$. **b** Close-up view of DCF binding site in OATP1B1-D. **c-e** Superimposition of OATP1B1-E$_{in}$ and OATP1B1-D aligned by CTD. OATP1B1-E$_{in}$ is colored in gold and marine for NTD and CTD, respectively. OATP1B1-D is colored in gray. Upon binding of E3S/DCF, local adjustments were observed at the binding site (**c**), in TM8 (**d**) and TM7 (**e**). The adjustment of TM7 potentially triggers synergic movement of latch-like ICH. Specific residues of OATP1B1-E$_{in}$/D are shown as spheres and colored by green and yellow, respectively.
