# Supplementary material for: Cryo-EM structures of human organic anion transporting polypeptide OATP1B1
Source: Cell Res. 2023 Sep 6;33(12):940–51. doi: 10.1038/s41422-023-00870-8 (PMC10709409; doi:10.1038/s41422-023-00870-8)
Supplement: Supplementary file 26 — Supplementary information, Fig. S14 [file 41422_2023_870_MOESM26_ESM.pdf]

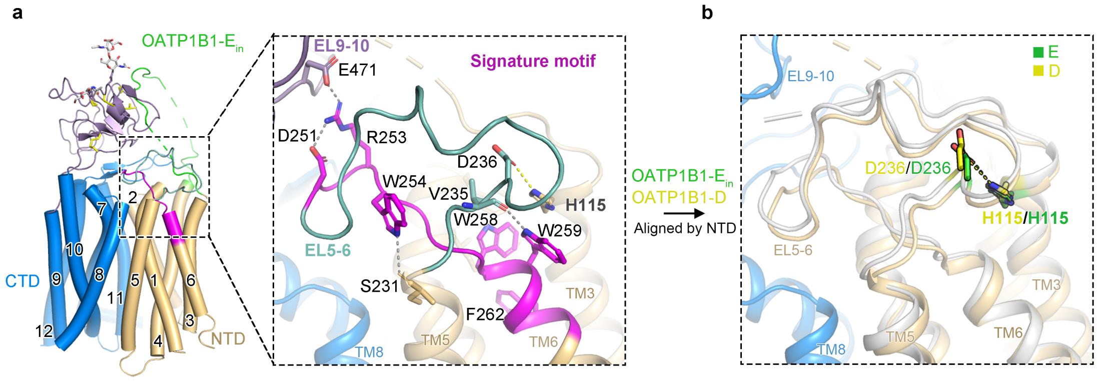

Supplementary information, Fig. S14 Direct interactions between H115 and D236 in both states of OATP1B1. a Overall architecture of inward-open OATP1B1 (left panel) and close-up view of signature motif spanning the border of EL5-6 and TM6 (right panel). The motif is highlighted in magenta. Key residues previously indicated to be in relation to transport activity and protein surface expression are shown as sticks. **b** OATP1B1-E_{in} and OATP1B1-D are aligned by NTD showing that conserved H115 forms electrostatic interaction with D236 in both inward-open and outward-open states.
