# Supplementary material for: Cryo-EM structures of human organic anion transporting polypeptide OATP1B1
Source: Cell Res. 2023 Sep 6;33(12):940–51. doi: 10.1038/s41422-023-00870-8 (PMC10709409; doi:10.1038/s41422-023-00870-8)
Supplement: Supplementary file 27 — Supplementary information, Fig. S15 [file 41422_2023_870_MOESM27_ESM.pdf]

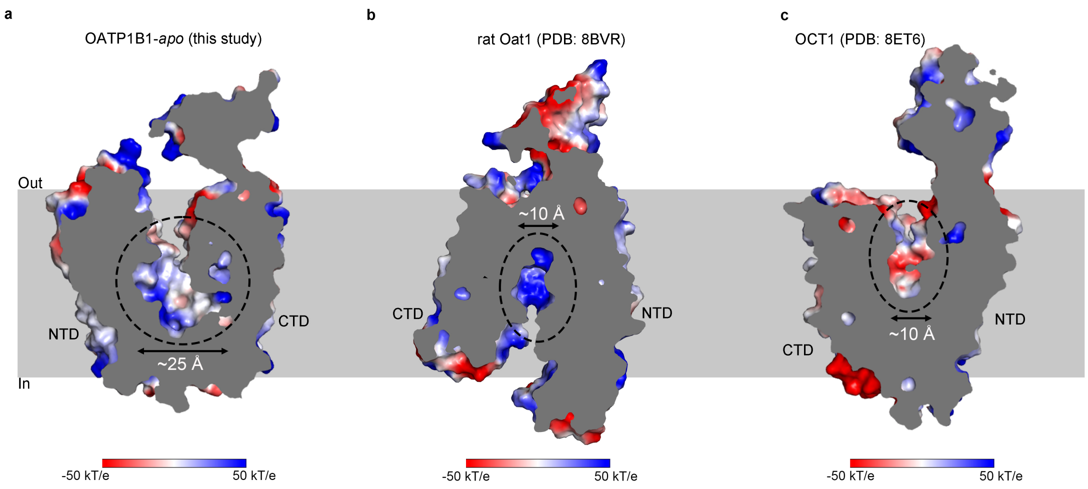

Supplementary information, Fig. S15 Substrate binding pockets in OATP1B1, rat Oat1 and OCT1. a The surface of OATP1B1-*apo* colored according to the calculated electrostatic potential shows hydrophobicity and electropositivity of the central cavity. **b-c** The structures of rat Oat1 (PDB: 8BVR) (**b**) and human OCT1 (PDB: 8ET6) (**c**) reveal electropositivity and electronegativity of the central cavity, respectively.
